# The Current Status and Evaluation of Artificial Intelligence in Bladder Cancer: Clinical Applications and Research

**DOI:** 10.34133/csbj.0197

**Published:** 2026-10-19

**Authors:** Zhuo Yin, Joshua W. K. Ho

**Affiliations:** ^1^ School of Biomedical Sciences, Li Ka Shing Faculty of Medicine, The University of Hong Kong, Hong Kong SAR, China.; ^2^ Department of Urology, The Second Xiangya Hospital of Central South University, Hunan, China.; ^3^ School of Biomedical Engineering, The University of Hong Kong, Hong Kong SAR, China.

## Abstract

Bladder cancer ranks as the 10th most common malignancy worldwide. Advances in technology are continuously reshaping its diagnosis, treatment selection, and prognosis assessment. In recent years, artificial intelligence (AI) has demonstrated marked potential in transforming bladder cancer care. By integrating and analyzing multi-source data, AI enhances the precision and efficiency of disease management. This review comprehensively examines the current applications of AI across various domains, including radiographic imaging analysis, pathological diagnosis, cystoscopic image processing, molecular subtyping, drug development, organoid research, prognostic modeling, and medical device innovation. It highlights the advantages of AI over conventional methods, outlines model development strategies, and summarizes commonly employed algorithms. Furthermore, we discuss the key challenges in translating AI tools into real-world clinical practice. This narrative review aims to inform the future development of AI-driven digital health systems, ultimately contributing to the realization of precision medicine and personalized management for bladder cancer patients.

## Introduction

Bladder cancer, one of the most prevalent malignancies of the urinary system, exhibits a rising global incidence. Recent statistics rank it as the 10th most common cancer worldwide [1]. The cornerstone of effective treatment lies in the precise determination of clinical stage, pathological grade, and molecular subtype. Conventionally, this crucial information is acquired through a combination of radiological imaging [e.g., computed tomography (CT), magnetic resonance imaging (MRI), and positron emission tomography–computed tomography (PET-CT)], cystoscopy, histopathological examination, and molecular biology techniques such as genomic sequencing. These diagnostic pillars, which are inherently image-centric or generate vast datasets, present ideal opportunities for the application of deep learning in medical image analysis, multi-omics data integration, and multimodal fusion. Moreover, artificial intelligence (AI) holds the potential to transcend the limitations inherent to individual clinicians’ experience and cognitive capacity. This review will elucidate these application scenarios with concrete examples.

Radiographic imaging plays a critical role in the clinical staging of bladder cancer, directly influencing treatment selection and prognostic evaluation. Traditional manual interpretation, which relies on radiologists’ subjective visual assessment, is often limited by inter-observer variability [2]. In contrast, AI, powered by deep learning models, can automatically extract multidimensional features from imaging data, thereby improving the accuracy of lesion detection, segmentation, and classification [2]. For instance, the integration of T2-weighted imaging (T2WI) with denoising deep learning reconstruction has been shown to enhance the diagnostic performance of the vesical imaging–reporting and data system (VI-RADS) in bladder cancer staging [3]. Furthermore, deep learning features extracted from pretrained ResNet18, when combined with machine learning (ML) classifiers, have demonstrated the ability to accurately grade bladder tumors [4].

Histopathological examination remains the gold standard for cancer diagnosis. AI-based computational pathology holds immense potential by enabling rapid, quantitative analysis of whole-slide image (WSI), facilitating objective assessments of tumor grade [5], stage [6], and immune cell infiltration [7]. Furthermore, AI models derived from digital pathology can predict recurrence and progression risks in non-muscle-invasive bladder cancer (NMIBC), forecast responses to Bacillus Calmette-Guérin (BCG) immunotherapy, and identify high-risk patients who may benefit from early radical cystectomy (RC) [8]. Exemplifying this advancement, a recently developed AI model for diagnosing lymph node metastasis (LNM) from WSI achieved an accuracy of 0.983 [95% confidence interval (CI), 0.941 to 0.998], surpassing the performance of both primary and senior pathologists [9].

Cystoscopy is indispensable for the initial diagnosis, prognostic evaluation, and post-operative surveillance of bladder cancer. However, its reliance on real-time visual inspection renders it susceptible to missing flat lesions like carcinoma *in situ* (CIS) [10,11], with diagnostic accuracy further influenced by image noise[12], illumination variability [11], and operator experience [13]. AI-augmented cystoscopy addresses these limitations by employing deep learning to analyze white-light cystoscopy (WLC) images, performing tasks such as image-level classification, lesion localization, precise segmentation, and digital staining to simulate enhanced imaging effects (e.g., blue-light cystoscopy) [14]. Representative systems include CystoNet [15], CystoNet-T [16], cystoscopy artificial intelligence diagnostic system (CAIDS) [17], and red, green, blue (RGB) video analysis systems [18].

Molecular subtyping is a vital tool for quantitatively analyzing the heterogeneity of bladder cancer. Transcriptomic and genomic profiling provide deep insights into the unique molecular characteristics and biological behaviors of tumors [19]. However, the high dimensionality, sparsity, and noise inherent in data like single-cell RNA sequencing (scRNA-seq) pose substantial barriers to clinical translation. Deep learning offers a promising solution to these challenges by effectively modeling complex relationships within single-cell and bulk transcriptomic data [20]. A prominent example is the development of an AI model that integrates mutational and gene expression data to predict responses to immune checkpoint inhibitors [anti-programmed cell death protein 1 (anti-PD-1)/programmed death-ligand 1 (anti-PD-L1)] in patients with advanced bladder cancer [21]. Additionally, emerging approaches that fuse computer vision (from histology) with genomic data enable powerful multimodal predictions of molecular subtypes and clinical outcomes [22].

Figure 1 delineates a multimodal integrated framework for AI in bladder cancer, which serves as the organizational blueprint for the subsequent sections of this review.

**Fig. 1.**
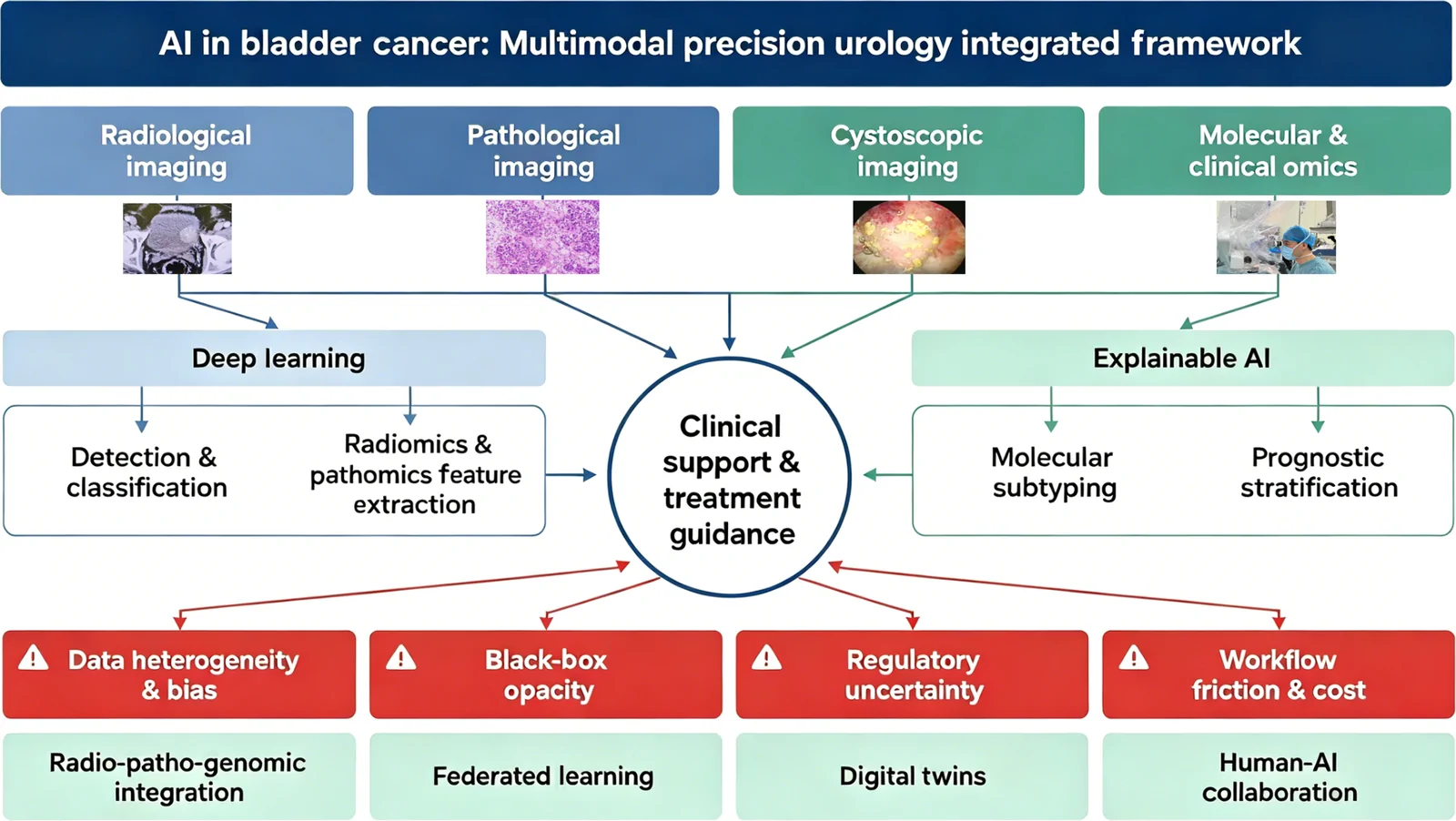
AI in bladder cancer: multimodal precision urology integrated framework.

This review was conducted as a structured narrative review, synthesizing key literature to provide a comprehensive overview of AI applications in bladder cancer. While we employed structured approaches to literature identification, independent screening, and data extraction to enhance transparency and reproducibility, this review was not designed to comprehensively capture every eligible publication. As such, it does not adhere to formal systematic review protocols (e.g., PRISMA) and should not be interpreted as an exhaustive evidence synthesis. This narrative review draws on published literature and selected preprints identified through a structured search of PubMed and Web of Science (2015–2025). We also browsed arXiv for relevant methodological advances. Search terms combined AI-related keywords (“artificial intelligence”, “machine learning”, “deep learning”, “radiomics”, “neural networks”) with bladder cancer terms (“bladder cancer”, “urothelial carcinoma”, “cystoscopy”, “pathology”, “radiology”, “molecular subtyping”, “prognosis”, “drug development”). We limited the search to English-language peer-reviewed original articles, systematic reviews, and preprints that had been externally validated. Conference abstracts without full text, editorials, commentaries, and studies lacking quantitative performance data were excluded. Both authors independently screened titles and abstracts, resolving disagreements through discussion.

To further characterize the maturity of included studies, we adapted the Food and Drug Administration (FDA) 3-phase evidence framework for AI/ML-based medical devices (analytical validation → clinical validation → clinical utility), stratifying analytical validation into internal (level I) and external/multicenter (level II) validation, and incorporating observer-based assessment (reader studies) as a distinct level III, given its critical role in evaluating AI-assisted diagnostic workflows. This resulted in a 5-level evidence hierarchy: level I (technical feasibility with internal validation only), level II (external or multicenter retrospective validation), level III (observer-based assessment/reader studies), level IV (prospective clinical validation), and level V (randomized implementation or clinical trials). Notably, no studies satisfying level V criteria were identified in our review; accordingly, Fig. 2A displays the evidence distribution for levels I to IV only. This hierarchy aligns with the Checklist for Artificial Intelligence in Medical Imaging (CLAIM) and the Consolidated Standards of Reporting Trials–Artificial Intelligence (CONSORT-AI) extension recommendations for translational AI research. The evidence level classification of studies included in this article was conducted according to this framework. Both authors then independently assigned each included study to the appropriate evidence level, with disagreements again resolved through discussion.

We conducted evidence-level assessment and visualization of the literature on AI models directly applicable to bladder cancer analysis. Among the 52 included studies, AI applications spanned 7 research fields: tumor imaging (*n* = 15), tumor pathology (*n* = 13), molecular subtyping (*n* = 10), general methodology (n = 4), cystoscopy (*n* = 4), prognosis prediction (n = 2), and others (*n* = 4) (Fig. 2B). The evidence levels were heavily skewed toward lower tiers, with 59.6% (31/52) at level I (internal validation only), 26.9% (14/52) at level II (external/multicenter retrospective validation), 9.6% (5/52) at level III (reader studies), and only 3.8% (2/52) at level IV (prospective validation), and no study reached level V (randomized trials) (Fig. 2D). Notably, all general-purpose models remained at level I, whereas 55.3% (21/38) of bladder cancer-specific models reached level II or higher, suggesting that domain-specific applications demonstrate more mature validation trajectories than universal foundation models. The detailed evidence classification for each study is provided in Supplementary Table S1 (Summary of evidence levels for bladder cancer AI literature).

**Fig. 2.**
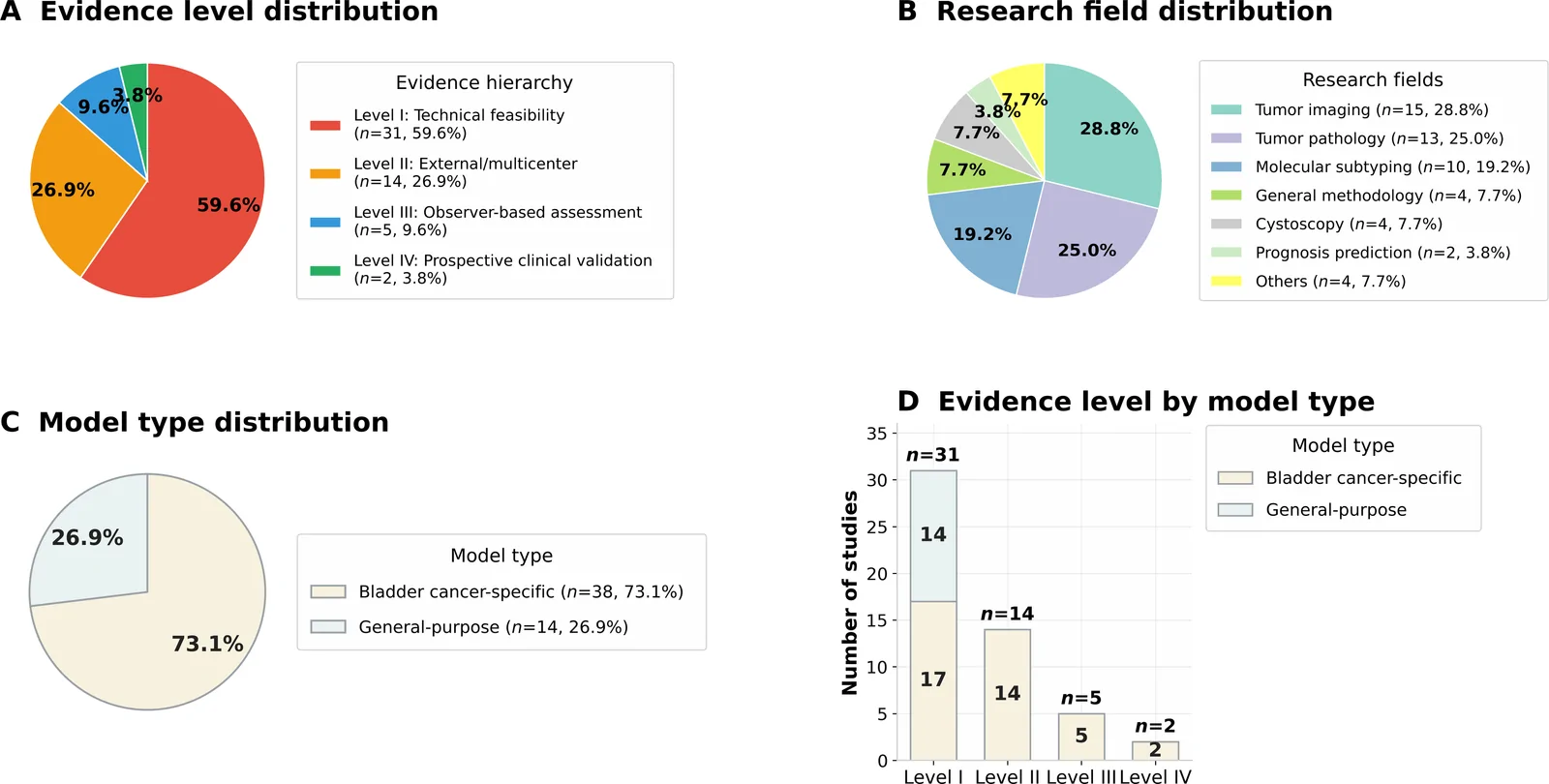
Evidence level and research field distribution for bladder cancer AI literature.

## Application of AI in Bladder Cancer Radiological Image Analysis

### The advantages of combining AI with traditional methods

CT and MRI are important for staging bladder cancer clinically. A study [23] shows that CT has relatively low overall accuracy in distinguishing NMIBC (≤pT1) from muscle-invasive bladder cancer (MIBC) (≥pT2). The overall accuracy for CT to correlate with final pT stage at RC ranges from 40% to 92%. In contrast, MRI has a sensitivity of 87% and a specificity of 79%. Combining MRI with the VI-RADS scoring system improves sensitivity and specificity [23]. However, VI-RADS relies on multiparametric magnetic resonance imaging (mpMRI), which includes T2WI, diffusion-weighted imaging (DWI), and dynamic contrast-enhanced (DCE) imaging [2]. The DCE sequence is associated with a prolonged acquisition time, susceptibility to motion artifacts, and interference from urine signal, along with a potential risk of contrast agent deposition—particularly in patients with renal insufficiency [2]. To address these limitations, a deep vesical imaging network (ViNet) model was developed for the detection of MIBC using high-resolution T2WI as an alternative to conventional mpMRI [2]. Moreover, deep learning-based reconstruction techniques have been shown to substantially reduce acquisition time for T2-weighted sequences while improving image quality [24]. Additionally, image reconstruction methods based on compressed sensing or AI algorithms lower the technical and hardware requirements for MRI examinations. This advancement enables institutions with limited resources—such as those unable to acquire high-field (e.g., 3.0 T) MRI systems—to enhance their clinical capacity for diagnosing and staging bladder cancer [25].

### Construction and application of AI models for radiological imaging

The construction of an AI model for radiological imaging of bladder cancer includes the following steps: data collection and preprocessing, feature extraction, and model training and validation [2,26]. Feature extraction is a key part of the construction process and includes 2 categories: handcrafted features and deep learning features [27]. Handcrafted features include morphological features (e.g., cancer volume and shape irregularity), texture features, and functional features [27]. The deep learning features are automatically learned through discriminative spatial-semantic feature extraction module [28], and they have the capacity to capture more complex image patterns. Commonly used algorithms encompass convolutional neural networks [e.g., 3-dimensional residual network (3D ResNet), visual geometry group (VGG), and U-Net] [2,29,30], 3D Vision Transformers [31], as well as traditional ML models like support vector machines (SVMs) and random forests [32,33]. Furthermore, to enhance performance, clinical prior knowledge is often incorporated into deep neural networks [29].

The mpMRI–computer-aided diagnosis (CAD) system [26] uses an innovative method of isometric surface analysis for feature extraction. It achieves precise feature capture by calculating the distance from points within the tumor to its boundary in order to construct nested surface layers. For bladder cancer T1/T2 staging, the system achieves an accuracy of up to 95.24% and an area under the receiver operating characteristic curve (AUROC) of 0.9864. Its performance is statistically significantly superior to that of comparative models, such as random forest and residual network with 50 layers (ResNet50).

The ViNet model [2] achieved successful diagnostic performance with an AUROC of 0.87 using a single high-resolution T2WI sequence. ViNet even outperformed mpMRI-based models. The model’s ability to extract rich information from a single sequence is enabled by leveraging self-supervised learning, no new U-Net (nnU-Net) segmentation localization, and attention mechanisms. This allows for safer, faster, and more cost-effective AI diagnosis, eliminating the need for contrast agents.

### Systematic comparison between mpMRI-CAD and ViNet

Although both ViNet [2] and mpMRI-CAD [26] demonstrate promising performance in MRI-based bladder cancer staging, direct quantitative comparison is precluded by substantial heterogeneity in methodology, validation design, and clinical objectives. Table 1 summarizes the key differences between the 2 models.

**Table 1.** Methodology and performance comparison between mpMRI-CAD and ViNet

Aspect	mpMRI-CAD [26]	ViNet [2]
Technical core	Iso-surface analysis with nested feature extraction	Self-supervised foundation model + nnU-Net attention mask
Input sequences	T2WI + DWI (ADC)	Single-sequence hrT2WI (contrast-free, including both FS and non-FS)
Segmentation	3D CNN + adaptive shape prior	nnU-Net (automatic, bladder wall + tumor)
Feature extraction	Handcrafted features Texture (GLCM/GLRLM) + functional (ADC CDF) + morphological	SSL-pretrained deep features + attention-guided refinement
Sample size	Total sample size: 42 cases (single-center)	Total sample size: 491 cases (3 centers retrospective cohort) Training/test split: Center 1 was randomly divided into training set (312) + internal test set (79) External validation: Center 2 (*n* = 44) + Center 3 (*n* = 56) = 100 cases Pretraining scale: The encoder was self-supervised pretrained on >40,000 cross-modal images
Class Imbalance	Category distribution: 21 cases of T1 stage, 21 cases of T2 stage Balance: Perfectly balanced (1:1) Limitation: This balanced allocation does not reflect the natural distribution of T1/T2 in the real world, limiting generalizability	Category distribution: Training set: 108 MIBC (34.6%) vs. 204 NMIBC (65.4%) Internal test: 27 MIBC (34.2%) vs. 52 NMIBC (65.8%) External validation: 38 MIBC (38.0%) vs. 62 NMIBC (62.0%) Imbalance level: Approximately 1:2 (NMIBC is about twice the size of MIBC) Potential impact: Recall for the minority class (MIBC) may be low
Validation strategies	Main method: Leave-one-subject-out (LOSO) Evaluation metrics: ACC 95.24%, SEN 95.24%, SPE 95.24%, AUROC 0.9864 Limitations: LOSO may produce biased accuracy estimates, lack of an independent external test set, and limited generalizability	Main method: Multicenter external validation (strict split) Internal testing: Internal test set of center 1 (*n* = 79) External validation: Center 2 (*n* = 44) + Center 3 (*n* = 56) OR values: Internal validation: ViNet vs. ablation model OR 7.41 vs. 1.85–2.70 (all *P* < 0.05) External validation: ViNet vs. ablation models OR 1.31–3.89 vs. 0.89–9.75 (*P* = 0.03–0.15) Clinical validation: Comparison of performance of 6 readers using VI-RADS versus ViNet-adjusted VI-RADS MIBC missed by VI-RADS 2: ViNet upgraded in 62.9% (17/27) NMIBC overestimated by VI-RADS 3–5: ViNet downgraded in 84.1% (69/84), 62.5% (35/56), and 67.9% (19/28) Limitations: Retrospective design, variability in external validation, and prospective studies are still needed to verify stability

ACC, accuracy; ADC, apparent diffusion coefficient; CDF, cumulative distribution function; GLCM, gray-level co-occurrence matrix; GLRLM, gray-level run-length matrix; SEN, sensitivity; SPE, specificity

Methodologically, mpMRI-CAD employs iso-surface analysis to extract texture, functional, and morphological features from nested tumor layers, assuming that depth-dependent histological variation carries diagnostic value. In contrast, ViNet leverages a self-supervised foundation model pretrained on >40,000 cross-modal datasets, with nnU-Net-generated attention masks guiding the model to focus on MIBC-relevant regions (tumor–basal interface), thereby enhancing interpretability.

In terms of performance, mpMRI-CAD reported superior internal metrics (AUROC 0.9864) using leave-one-out cross-validation on 42 single-center patients. ViNet achieved moderate but more generalizable performance (internal AUROC 0.87, external AUROC 0.75) on a large multicenter cohort (*n* = 491), operating on contrast-free hrT2WI. Both models have been rigorously benchmarked against multiple alternatives—mpMRI-CAD against Random Forest (RF), SVM, and ResNet50, and ViNet against radiomics and simpler ResNet variants—consistently showing superior performance in their respective validation settings.

Clinically, mpMRI-CAD prioritizes maximal diagnostic precision in controlled settings, while ViNet emphasizes generalizability and patient safety (no gadolinium, faster acquisition, broader applicability across centers). Future studies should perform head-to-head validation on a shared public dataset using standardized VI-RADS-stratified metrics.

### AI applications in staging, nodal assessment, and prognostic stratification

Beyond the construction of dedicated staging models such as mpMRI-CAD and ViNet, AI has been increasingly applied to broader clinical tasks—including preoperative staging, LNM prediction, and prognostic stratification—using diverse imaging modalities and methodological innovations.

#### Staging and histological grade prediction

Early efforts in AI-based bladder cancer staging focused on integrating ML into conventional imaging workflows. Garapati *et al*. [34] pioneered an automated CT urography analysis pipeline that linked tumor segmentation directly to T-stage classification, reducing operator dependence while maintaining diagnostic accuracy. As deep learning matured, Yang *et al*. [35] transitioned from handcrafted radiomics to end-to-end convolutional neural networks, directly learning hierarchical spatial features from CT images to noninvasively differentiate MIBC from NMIBC without manual feature engineering. Recognizing that prognostication benefits from multimodal data fusion, Song *et al*. [4] subsequently developed a deep learning radiomics nomogram that integrated handcrafted radiomics and deep learning features from CT images with clinical indicators, enabling preoperative prediction of bladder cancer pathological grade. More recently, Xiong *et al*. [36] conducted a systematic comparative analysis demonstrating that hybrid models combining quantitative radiomic signatures with clinical variables markedly outperform either modality alone in T-stage prediction. To address intratumoral heterogeneity—a limitation of whole-tumor analysis—Du *et al*. [37] introduced a habitat-based radiomics framework that, after initial texture feature extraction, clusters contrast-enhanced CT tumor subregions using K-means to identify distinct intratumoral habitats, followed by habitat-specific radiomic feature extraction. This approach captured heterogeneity invisible to conventional whole-tumor analysis in a multicenter setting, achieving superior performance (external test AUROC = 0.825) compared to uniphase and multiphase models. Lisson *et al*. [38] identified imaging timing relative to transurethral resection of the bladder (TURB) as a critical variable for model stability, showing that CT performed more than 14 d after TURB yields superior ML performance in distinguishing T2 from T3 disease compared to CT performed within 14 d post-TURB. This finding underscores the necessity of standardized acquisition protocols with respect to post-surgical timing to ensure AI reproducibility.

#### LNM prediction

Accurate preoperative identification of LNM is essential for determining the extent of pelvic lymphadenectomy and neoadjuvant chemotherapy eligibility. To overcome the limited sensitivity of conventional size-based CT criteria, Girard *et al*. [39] combined morphological and metabolic quantitative features from [^18^F]fluorodeoxyglucose (FDG) PET/CT [including lymph node (LN) maximum standardized uptake value (SUVmax), LN size product, and primary tumor size product] within a random forest classifier to detect pelvic LN involvement in MIBC. The model achieved an AUROC of 0.59 in the validation set, which was not statistically significantly different from a consensus of expert radiologists (AUROC = 0.64, *P* = 0.54), with equal interrater agreement (Kappa = 0.66). This suggests that a simple quantitative feature-based ML approach can perform comparably to expert interpretation, although diagnostic accuracy remains modest. Gresser *et al*. [40] compared manual versus automated segmentation for CT-based radiomics nodal staging in bladder cancer, demonstrating that the manual segmentation-based radiomics signature achieved superior diagnostic accuracy (AUROC = 0.80) compared to the fully automated approach (AUROC = 0.70), while a combined model incorporating both radiomics and radiologist assessment yielded an AUROC of 0.81. Extending these approaches through deep learning, Sun *et al*. [41] established a CT-based combined model incorporating handcrafted radiomics features, deep learning features, and clinical predictors, which achieved robust prediction of LNM in bladder cancer patients across 2 independent centers (external test set AUROC = 0.834). The model also demonstrated satisfactory risk stratification for progression-free survival and good clinical utility according to decision curve analysis.

#### Prognostic assessment and treatment response monitoring

AI-based imaging analysis has gradually shifted from anatomical staging toward individualized survival prediction and therapy response assessment. Cha *et al*. [42] provided an early demonstration of this potential by combining radiomics with deep learning to quantitatively assess bladder cancer treatment response on CT, integrating high-throughput handcrafted features with hierarchical deep representations to provide an objective imaging biomarker for chemotherapy efficacy evaluation. Expanding beyond tumor-centric analysis, Ying *et al*. [43] applied automated AI segmentation to quantify skeletal muscle volume on preoperative CT, identifying sarcopenia as an imaging-derived independent predictor of post-cystectomy survival—thereby broadening AI’s role into body-composition-based frailty assessment. More recently, Wei *et al*. [44] developed a multicenter CT deep learning model that directly extracts prognosis-associated imaging phenotypes to predict overall survival following RC, outperforming traditional clinical staging systems in prognostic discrimination.

### Challenges in the clinical translation of AI in radiological imaging

Several key challenges remain to be systematically addressed in the research and clinical translation of AI in medical imaging. First, the heterogeneity of imaging data poses a substantial obstacle. Variations in imaging modalities and scanning parameters lead to differences in image reconstruction protocols, which directly affect the stability of radiomics feature extraction. This weakens the reproducibility and generalizability of models [45,46]. Second, concerns have been raised about the adequacy of clinical validation. Among 516 studies on AI-based medical image diagnosis, only 6% (31 studies) underwent external validation. None of the 31 studies adopted all 3 design features for external validation: diagnostic cohort design, multi-institutional inclusion, and prospective data collection [47].

The lack of assessment of performance variability is an issue that cannot be ignored. Among the 221 medical image segmentation papers accepted at the 2023 Medical Image Computing and Computer Assisted Intervention (MICCAI) conference, only 0.5% (1 paper) reported confidence intervals to reflect performance uncertainty; more than half of the papers did not evaluate performance variability. This reporting approach that neglects external uncertainty leaves us without sufficient evidence to support which models can be translated into clinical practice [48].

Additionally, technical integration and data flow management pose practical challenges. Future efforts must deeply integrate automated processing workflows, such as the Extensible Neuroimaging Archive Toolkit (XNAT) and Digital Imaging and Communications in Medicine (DICOM) image analysis and archiving systems, with existing healthcare infrastructure. This integration would reduce data retrieval latency and enable real-time AI analysis of images, as well as secure transmission to portable devices under de-identification [49].

To provide a systematic overview of the key studies discussed throughout the Application of AI in Bladder Cancer Radiological Image Analysis section, we summarize in Table 2 the main characteristics and findings of representative AI applications in bladder cancer radiological image analysis, organized by first author, imaging modality, task type, core methodology, and reported performance.

**Table 2.** Summary of key studies on AI applications in bladder cancer radiological image analysis

First author	Year	Modality	Task	Core method	Key performance/finding
Gong *et al*. [2]	2025	MRI (hrT2WI)	MIBC vs. NMIBC	ViNet (SSL + nnU-Net)	AUROC 0.87 (internal), 0.75 (external); contrast-free
Hammouda *et al*. [26]	2021	mpMRI (T2WI + DWI)	T1/T2 staging	mpMRI-CAD (iso-surface analysis)	Accuracy 95.24%, AUROC 0.9864
Garapati *et al*. [34]	2017	CT	T-stage classification	Automated CT urography pipeline	Reduced operator dependence
Yang *et al*. [35]	2021	CT	MIBC vs. NMIBC	End-to-end CNN	Direct differentiation without handcrafted features
Song *et al*. [4]	2023	CT	Pathological grade	DL radiomics nomogram	Integrated radiomics + clinical indicators
Xiong *et al*. [36]	2024	CT	T-stage prediction	Hybrid radiomics-clinical model	Outperformed either modality alone
Du *et al*. [37]	2025	CT	Muscle invasion prediction	Habitat-based radiomics	Captured intratumoral heterogeneity
Lisson *et al*. [38]	2025	CT	T2 vs. T3 (post-TURB timing)	ML radiomics with imaging timing stratification	Delayed post-TURB imaging (>14 d) improved stability
Girard *et al*. [39]	2023	[18F]FDG PET/CT	LNM prediction	ML (morphological + metabolic)	Kappa = 0.66 (agreement with expert consensus)
Gresser *et al*. [40]	2023	CT	Nodal staging	Manual vs. auto segmentation	Manual segmentation outperformed auto-segmentation
Sun *et al*. [41]	2024	CT	LNM prediction and prognosis	Deep learning radiomics	Robust across 2 centers
Cha *et al*. [42]	2017	CT	Treatment response	Radiomics + DL	Objective biomarker for chemotherapy
Ying *et al*. [43]	2021	CT	Post-cystectomy survival	Automated sarcopenia quantification	Independent predictor of survival
Wei *et al*. [44]	2024	CT	Overall survival	Multicenter DL model	Outperformed clinical staging

MIBC, muscle-invasive bladder cancer; NMIBC, non-muscle-invasive bladder cancer; LNM, lymph node metastasis; SSL, self-supervised learning; DL, deep learning; ML, machine learning; CECT, contrast-enhanced computed tomography

## AI in Bladder Cancer Pathology

### Advantages of AI-assisted pathological diagnosis

AI-based pathological analysis departs from the traditional microscope-first workflow. While the following 5 gains are often cited as transformative, a critical examination reveals that each advantage comes with conditional caveats that currently limit its real-world impact.

First, algorithms are often described as intrinsically objective and reproducible [50]. However, this objectivity holds only under strictly controlled conditions (same scanner, same digitization protocol, same model version). Reproducibility across different laboratories remains a major challenge due to domain shift and batch effects, which are rarely systematically evaluated in the literature.

Moreover, this consistency is paired with dramatic gains in throughput and efficiency. Yet, the reported “seconds-per-slide” inference time [51] rarely accounts for the rate-limiting step in clinical workflows: WSI digitization (which takes minutes) and manual quality control (QC). Furthermore, most published efficiency benchmarks use curated datasets, not real-world messy slides containing artifacts, folds, or out-of-focus regions.

At the same time, the software performs genuine quantitative analysis, measuring subvisual features that the human eye cannot resolve [52]. Critically, many of these “novel morphometric features” lack prospective validation as independent biomarkers. There is a substantial risk of overfitting to training cohorts, and their biological meaning often remains an uninterpretable “black box”, limiting pathologist trust.

These rich measurements feed new prognostic models beyond tumor, node, metastasis (TNM) stage [53]. While promising for biomarker discovery, the majority of these models have not been validated in prospective clinical trials.

Finally, AI acts as a vigilant second reader, cutting miss rates [6]. However, no large-scale randomized controlled trial has yet demonstrated that AI-assisted second reading improves patient outcomes in bladder cancer specifically.

### Construction of pathology AI models and comparison with representative models

A critical comparison of current pathology foundation models (Table 3) reveals a marked disconnect between reported technical performance and real-world clinical readiness. While models like UNI [54] and GigaPath [55] achieve state-of-the-art (SOTA) on retrospective benchmarks, their limitations in generalizability, hardware accessibility, and validation rigor are often underemphasized.

**Table 3.** Comparison of current major pathology foundation models, and whether they have been applied to bladder cancer. All models were evaluated using internal benchmark datasets (level I evidence); none have undergone independent external validation specifically for bladder cancer (level II or higher).

Model name	Publisher/team	Core architecture and features	Data scale	Takeaway	GPU need	Applied to bladder cancer?
UNI [54]	Harvard Medical School/Mahmood Lab	Architecture: Vision Transformer-Large (ViT-L). Pretraining Strategy: DINOv2 self-supervised (label-free masked modeling)	WSI Count: 100,426 Coverage: 20 organ types	Strong few-shot performance; lacks text encoder or multimodal interface. Generic, not bladder-tuned.	16 GB	No bladder-specific benchmark; included in multi-cancer evaluation
CONCH [56]	Harvard Medical School/Mahmood Lab	Three-tower vision-language foundation model: ViT image encoder + Transformer text encoder + CoCa fusion decoder	1.17 million image-caption pairs	Effective zero-shot transfer on multiple cancers. High VRAM requirement. No native WSI-level reasoning.	24 GB	No bladder-specific benchmark in original work; external studies have explored urothelial carcinoma zero-shot classification
BEPH [59]	Shanghai Jiao Tong University, China	BEiT-v2 masked image modeling (MIM). ViT-Base backbone + lightweight MLP head	WSI Count: 11,760 Coverage: 32 cancer types	Compact and interpretable; achieves strong performance with limited labeled data. Restricted to TCGA, limiting real-world generalizability.	16 GB	Evaluated on BLCA, though not the primary focus
PathOrchestra [58]	SenseTime Med/Air Force Medical University/Tsinghua	300M parameters, multimodal	WSI Count; 287,424 Bladder task: 324 WSI + 112 ROI	Handles Chinese pathology reports; supports zero-shot bladder classification. Trained on fewer WSIs than contemporaneous large-scale models.	V100-level	Supports automated report generation; achieved >95% accuracy on internal bladder cancer screening set
GigaPath [55]	Microsoft Research/University of Washington/ Providence	DINOv2 self-supervised pretraining. LongNet sparse attention processes entire slide sequences (slide-level)	WSI count: 171,189	Whole-slide modeling: No subsampling, preserves global context Zero-shot mutation prediction (FGFR3, BRCA1/2, etc.)	22 GB	No bladder-specific benchmark; mutation prediction demonstrated for EGFR
PRISM2 [57]	Paige	Uses original perceiver architecture. BioGPT-Large as text base.	WSI count: 2,350,518 Specimens: 685,507	Commercial platform; proprietary WSI-level encoder. Limited public bladder data. High memory requirements.	24 GB	Bladder cancer not highlighted in original reports

ViT-L, Vision Transformer-Large; WSI, whole-slide image; ViT, Vision Transformer

For example, the UNI model [54] demonstrates impressive zero-shot cross-cancer generalization. However, its training data (Mass-100K) originate almost exclusively from 2 academic medical centers [Massachusetts General Hospital (MGH) and Brigham and Womenʼs Hospital (BWH)]. No systematic evaluation of UNI’s performance on bladder cancer slides from community hospitals with variable tissue fixation protocols and digital slide scanners has been published (a domain-shift risk elaborated in the next section). The same problem existed in the cross-modal optimization for natural clinical histology (CONCH) model [56].

From a reproducibility perspective, PRISM2 [57] has not publicly released its training code or model weights, which limits independent verification of its reported results and raises concerns regarding FAIR principles. By contrast, PathOrchestra [58] has made its model weights and inference code publicly available. Nevertheless, the hardware barriers for model training and deployment remain prohibitively high. PathOrchestra’s pretraining required 4 nodes × 8 A100 80 GB graphics processing units (GPUs) (32 A100 80 GB GPUs in total), far beyond the capacity of typical hospital pathology departments and making these models currently infeasible for routine clinical deployment.

A more subtle but critical issue is evaluation bias: Almost all models compare themselves against weak baselines (e.g., ResNet-50) rather than against SOTA task-specific models or, more importantly, against expert pathologists under realistic clinical conditions (time pressure, incomplete clinical history, variable slide quality). Consequently, the “SOTA” claims in Table 3 should be interpreted as technical benchmarks on curated data, not as evidence of clinical superiority.

### Domain-specific barriers to bladder cancer translation

The critical comparison in Table 3 reveals a marked disconnect between the technical sophistication of current pathology foundation models and their readiness for bladder cancer diagnostics. A structured evidence appraisal of the 13 studies evaluated in this review ([5–9], [54–59], [60,61]) using a 5-level hierarchy underscores this gap: All foundation model papers were classified as level I (technical feasibility with internal validation only), with none achieving level II (external or multicenter retrospective validation) or higher specifically for bladder cancer applications. This uniform absence of bladder-specific external validation raises fundamental questions about whether benchmark performance on retrospective, curated datasets translates to real-world clinical environments.

Four domain-shift factors merit particular emphasis. First, scanner variability represents a largely unquantified risk. Foundation models such as UNI [54] and GigaPath [55] were trained and benchmarked on slides digitized with specific scanner models under controlled laboratory conditions. Bladder cancer transurethral resection specimens, however, frequently contain blood, necrotic debris, and cautery artifact that exacerbate scanning heterogeneity—glare, variable focus, and contrast inconsistencies that patch-level feature extractors may misinterpret as tissue architecture. The modest discriminative performance of existing bladder-specific models [slide-based lymphovascular invasion predictor (SBLVIP) AUROC 0.74, 95% CI 0.55 to 0.83 in the Renmin Hospital of Wuhan University (RHWU) cohort and AUROC 0.74, 95% CI 0.58 to 0.89 in the People’s Hospital of Hanchuan City (PHHC) cohort [60]] may partly reflect uncontrolled scanner domain shifts between training and validation settings, rather than intrinsic biological limitation.

Second, staining heterogeneity poses an acute challenge for bladder cancer diagnosis. The distinction between high-grade papillary urothelial carcinoma and CIS relies critically on nuclear pleomorphism assessment, which is exquisitely sensitive to hematoxylin staining intensity. Variations in eosinophilia across laboratories further obscure the tumor–stromal interface—a feature essential for determining muscularis propria invasion. Current foundation models offer no built-in staining normalization; their reliance on massive pretraining datasets ([54]: 100,426 WSIs; [55]: 171,189 WSIs; [57]: 2,350,518 WSIs) assumes that stochastic diversity in training data will confer robustness. Yet, these corpora are dominated by breast, lung, and gastrointestinal cancers, with bladder cancer representing a morphologically distinct minority that standard benchmark averages obscure.

Third, institutional differences in tissue processing amplify domain shift. Transurethral resection of bladder tumor (TURBT) specimens often experience delayed fixation compared with RC specimens, producing variable autolytic changes that alter nuclear detail. Slide thickness variations (3 μm versus 4 μm) directly affect the apparent depth of muscularis propria invasion—a parameter that determines the critical distinction between non–muscle-invasive and muscle-invasive disease. None of the foundation models in Table 3 were trained with explicit conditioning on tissue processing metadata, and their reported “zero-shot” generalization [54,55] has not been validated across institutions with divergent TURBT protocols.

Fourth, the histopathological complexity of bladder cancer challenges the homogenizing assumptions of general-purpose models. The 2022 World Health Organization (WHO) classification recognizes multiple aggressive variants (including nested, micropapillary, plasmacytoid, and sarcomatoid differentiation) that are clinically critical yet statistically rare. These variants are unlikely to be adequately represented in pretraining corpora optimized for common cancer types, creating a “long-tail” generalization failure.

### Future focus areas for pathology models

Despite the domain-specific barriers outlined in the previous section, emerging research directions [including unsupervised learning, molecular subtyping, and lymphovascular invasion (LVI) prediction] hold promise for addressing the validation gap. However, 3 fundamental methodological limitations must be prioritized in future investigations.

Widespread data bias and insufficient external validation are commonly observed across computational pathology research. Representative vision-language biomedical models such as Large Language and Vision Assistant for Medicine (LLaVA-Med) [62] are trained on heterogeneous public biomedical datasets, yet lack rigorous multi-center histopathological validation, standardized staining harmonization, and cross-scanner robustness assessment, thereby restricting their direct translation to routine bladder cancer diagnostics. In parallel, the SBLVIP model showed modest discriminative performance and broad confidence intervals. An AUROC of 0.74 (95% CI 0.55 to 0.83) in the RHWU cohort and an AUROC of 0.74 (95% CI 0.58 to 0.89) in the PHHC cohort [60]. Few models have undergone head-to-head comparison with expert pathologists under real-world clinical constraints, which complicates definitive conclusions regarding their independent diagnostic value in daily practice.

Another aspect that cannot be overlooked is the validity of the comparison methods. In a study on subtyping bladder tumors, the per-class AUROC values were as follows: basal type (0.89 ± 0.04), luminal type (0.88 ± 0.06), luminal p53-like type (0.89 ± 0.04), and double-negative type (0.76 ± 0.12) [63]. The authors argue that deep learning models can predict molecular subtypes of MIBC patients using only hematoxylin and eosin (H&E) slides, with performance superior to that of pathologists. However, the results of the study need to be interpreted cautiously. The 4 pathologists compared with AI were evaluated based on 186 randomly selected tiles from 36 patients, resulting in low overall accuracy. This is a sampling-based comparison, and the results may vary due to sampling differences. Additionally, when the same pathologists were later presented with WSIs supplemented with class activation map (CAM)-derived feature guidance, their accuracy increased by >20 percentage points [63]. This comparison is not entirely fair because the improvement confounds the effect of whole-slide context with the effect of algorithmic feature hints, and it suggests that the model may not necessarily outperform pathologists in real clinical practice where such algorithmic guidance is unavailable. This methodological discrepancy—evaluating pathologists on isolated tiles while evaluating the model on whole-slide context—illustrates a broader domain-shift problem: Patch-level or region of interest (ROI)-based model evaluation systematically underestimates the spatial contextual reasoning that pathologists employ for bladder cancer diagnosis, particularly for assessing architectural patterns relevant to variant histologies and invasion depth.

Finally, current pathology AI research is commonly constrained by limited high-quality multimodal datasets and overreliance on small, annotated retrospective cohorts, widening the translational gap between technical innovation and real-world clinical deployment. As demonstrated by recent vision-language foundation models in precision oncology [61], large-scale pretraining on unlabeled and unpaired pathological images and medical text enables robust cross-task generalizability without extensive manual annotation. Such multimodal integration uncovers complementary morphological and clinical information, addressing the narrow task specificity and limited external generalizability of conventional histology models.

## AI in Cystoscopic Image Analysis

### Shortcomings of traditional white light cystoscopy and improvement strategies

Existing evidence indicates that even experienced urologists exhibit limited sensitivity in detecting CIS during WLC [17,64,65]. Notably, CIS accounts for up to 94% of missed urothelial carcinomas [66]. Current strategies to improve the diagnostic performance of cystoscopy primarily focus on 2 approaches: enhancing visual contrast [67] and acquiring histological or molecular information [68]. A comparative summary of available image-enhancement cystoscopy techniques is provided in Table 4.

**Table 4.** A comparison of cystoscopy enhancement techniques: Principles, advantages, and limitations

Name	Principle	Advantages	Disadvantages
Blue-light cystoscopy (BLC) [175]	Intravesical instillation of a photosensitizer (e.g., 5-ALA or its derivative HAL) pre-operatively. Cancer cells selectively absorb and accumulate these substances, emitting red fluorescence under specific blue light wavelength.	Markedly improves detection rate for CIS and high-grade dysplasia, reducing omissions.	High false-positive rate (inflammation, recent BCG treatment can cause fluorescence), higher cost, requires special equipment.
Narrow band imaging (NBI) [176]	Filters white light, retaining only specific blue and green wavelengths. Hemoglobin strongly absorbs these, making the submucosal vascular network appear dark brown, creating sharp contrast with normal mucosa.	No drug instillation needed, real-time switching between WLC and NBI modes. More clearly displays abnormal cancer vasculature, improves cancer (especially papillary) detection rate, helps define cancer borders more precisely.	Strong dependence on operator experience, learning curve exists; sensitivity for some flat lesions (e.g., CIS) may be slightly lower than fluorescence cystoscopy.
CHROMA Imaging System [177]	Enhances contrast of vascular lesions through image processing, no exogenous contrast agent needed.	It can detect cancers missed under WLC.	Requires more research to validate clinical benefits.
Confocal laser endomicroscopy [178]	Provides real-time “optical biopsy” with cellular resolution via fiber optic probe.	Enables real-time assessment of histopathological features.	Expensive equipment, requires specialized training, not yet widely used. Although CLE shows some promise, it is not currently a reliable method for evaluating the bladder after bladder cancer surgery.
Optical coherence tomography (OCT) [67]	Analogous to “optical ultrasound”, uses near-infrared light for cross-sectional scanning of tissue, obtaining high-resolution real-time microstructural images (up to 2-mm submucosal).	Provides real-time *in vivo* pathological information, helps distinguish benign/malignant lesions, assess cancer stage (e.g., distinguish Ta/T1/T2).	Limited penetration depth, insufficient for overall assessment of large cancers, not yet a routine method.
Raman spectroscopy [179]	Identifies molecular-level abnormalities by analyzing energy changes from photon-tissue molecule interactions.	Can detect early molecular changes.	Technically complex, costly, still in research phase.
AI-augmented cystoscopy [14]	Uses deep learning models to analyze WLC images, enabling image classification, cancer localization, segmentation, digital staining.	No additional hardware/drugs needed, low cost, real-time diagnostic assistance.	Requires training data for continuous optimization.

Advantages of AI cystoscopy include improved detection rates [15], real-time feedback for enhanced efficiency [69], reduced interobserver variability, and improved diagnostic quality [15,70]. It also facilitates integration with other data modalities, increasing overall diagnostic accuracy [71].

### Construction of AI and representative models for cystoscopy

Creating AI models for cystoscopy faces unique challenges, including marked variations in image quality, inconsistent lighting conditions, and interference from specular reflections [11]. These technical issues need to be addressed in the preprocessing stage, including image enhancement, reflection removal, and motion artifact correction [67]. Cancer detection typically uses object detection algorithms [e.g., You Only Look Once (YOLO) and Faster Region-Based Convolutional Neural Network (Faster R-CNN)] [16], enabling real-time localization and classification of suspicious lesions. Cancer segmentation uses semantic segmentation networks (e.g., U-Net and DeepLab series) for pixel-level precise outlining [70,72].

CystoNet [15] was an early model. Its core mechanism uses a region-based convolutional neural network (R-CNN) for object detection. This model demonstrated that deep learning could assist real-time bladder cancer detection with high sensitivity (90.9%) and specificity (98.6%). This lays a solid foundation for the real-time application of AI in surgery and serves as the starting point for more complex subsequent models, such as CystoNet-T.

The CystoNet-T [16] mechanism combines local perception (CNN), global understanding (Transformer), and multi-scale analysis [feature pyramid network (FPN)]. Following feature extraction, one path feeds into the Transformer for global modeling and another into the FPN for multi-scale fusion. These 2 streams of information then converge to empower a precise object detector. This design enables CystoNet-T to handle vast differences in cancer size, morphology, location and background in cystoscopic images, achieving superior detection performance to that of traditional CNN models such as Faster R-CNN and YOLO.

CAIDS System [17] uses the pyramid scene parsing network (PSPNet). It comprises 3 modules, the core innovation of which is the pyramid pooling module. This module performs pooling at 4 different scales on the feature map to generate feature representations for different subregions. This allows the model to understand the image from multiple perspectives simultaneously: global (the entire image), semi-global (half the image), and local (small parts). Regardless of cancer size, the model can capture its contextual relationship with the surrounding environment, substantially improving the ability to identify small, flat lesions.

RGB Technology [18] takes the RGB image/video stream captured by the cystoscope and inputs it into a trained “object detection” or “semantic segmentation” deep learning model. The model then automatically localizes cancers and outlines their contours by analyzing pixel features in the image.

Figure 3 summarizes how the representative models work. As CystoNet-T is an upgraded version of CystoNet, the latter is not illustrated separately.

**Fig. 3.**
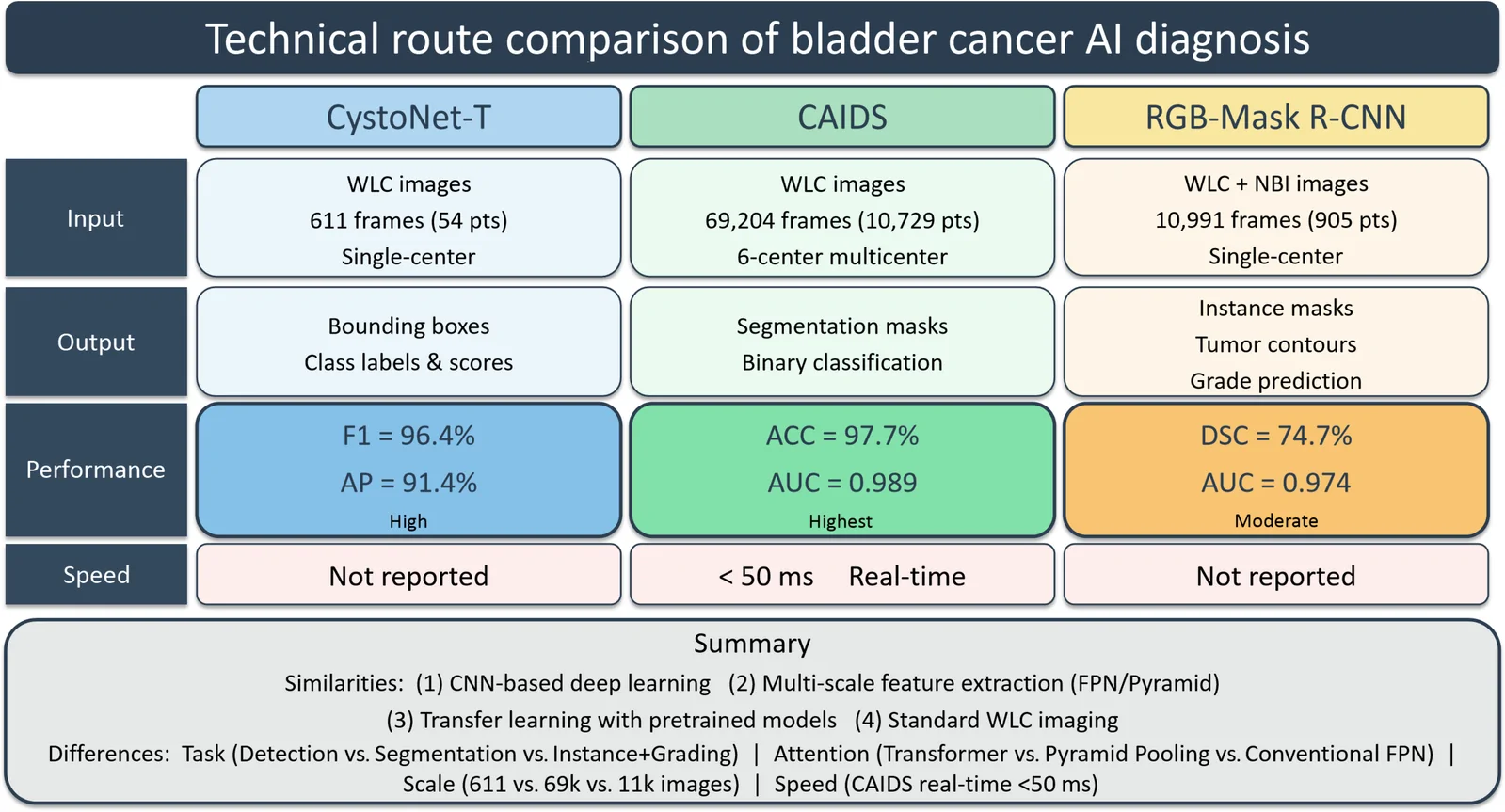
Technical router comparison of bladder cancer AI diagnosis.

### Comparison and methodological heterogeneity among models

The 4 models described—CystoNet [15], CystoNet-T [16], CAIDS [17], and RGB technology-based models [18]—adopt different technical paradigms for cystoscopic image analysis. Thus, a direct systematic quantitative comparison across these studies is not feasible due to several sources of heterogeneity.

First, the tasks differ: CystoNet and CystoNet-T perform object detection (bounding box localization), while CAIDS performs semantic segmentation (pixel-wise classification). RGB technology is applied to both tasks depending on the implementation. Detection and segmentation require different evaluation metrics [e.g., mean average precision (mAP) and sensitivity/specificity versus intersection over union (IoU)/Dice], making direct numerical comparisons inappropriate.

Second, datasets and annotation standards vary across studies. Each model was evaluated on different internal datasets without a public benchmark. Therefore, reported performance values—such as CystoNet’s sensitivity of 90.9% and specificity of 98.6%—cannot be fairly compared against other models under identical test conditions.

Third, methodological differences are substantial. CystoNet uses R-CNN region proposals; CystoNet-T integrates CNN, Transformer, and FPN for multi-scale feature fusion; CAIDS employs a pyramid pooling module to capture global-to-local context. These architectural choices have not been evaluated in a unified ablation or computational benchmark (e.g., inference speed and GPU memory).

In summary, no direct quantitative ranking among these models is possible from existing literature. Instead, they represent a technical progression addressing different clinical challenges. Future standardized evaluation should use public multi-center datasets, unified task definitions, and consistent metrics including average precision (AP) over IoU thresholds 0.5 to 0.95, F1-score, and inference speed.

### Characteristics and advantages of main models for image classification and semantic segmentation

Classifiers based on CNN such as CystoNet, ResNet50, VGG-19, VGG-16, InceptionV3, and Xception can achieve ≥90% sensitivity in the binary classification of cancerous versus normal mucosa [15,73]. The main factors leading to misjudgment are inflammation and bladder diverticula [15,73].

Encoding–decoding segmentation networks, such as U-Net [74] and HRNetV2 [65], achieve an mDice score of 0.80 to 0.85 on high-resolution frames. However, their performance drops sharply on low-resolution or motion-blurred frames, with an mDice score of 0.566.

The CNN + Vision Transformer (ViT) model with attention transformation achieved the highest accuracy (97.73%), surpassing the standalone CNN (94.32%) and ViT with full multi-head self-attention (MHSA) (95.12%) models [75]. These results suggest that the optimal balance between performance and computational efficiency is achieved by combining CNN for local feature extraction and ViT for global feature modeling while reducing attention complexity [75].

A comparative summary of the key characteristics and advantages of these models is provided in Table 5 (for image classification) and Table 6 (for semantic segmentation architectures).

**Table 5.** Comparison of image classification models

Model name	Core features	Main advantages	Typical medical tasks	Remarks
VGG19 [70]	Simple, regular structure	Easy to understand and implement, suitable for transfer learning	Classification	VGG19 model achieved 91.2% accuracy in cystoscopic image classification, distinguishing normal tissue, papilloma, flat, and mixed lesions. Now often a performance baseline
ResNet-34 [81]	Residual connections, solves vanishing gradient	Enables very deep networks, powerful performance	Classification, detection	One of the most mainstream backbones, balances performance and efficiency. The accuracy rate for bladder cancer identification during cystoscopy is 94.6%.
EfficientNetB0 [180]	Compound scaling optimization	High parameter efficiency, high accuracy	Classification	Preferred choice when computational resources are limited. The EfficientNetB0 model achieved an accuracy rate of 81% for bladder cancer.
Dense convolutional network (DenseNet) [181]	Dense connections, feature reuse	Few parameters, strong feature fusion capability	Classification (performs best on small datasets)	Unlike ResNets, which combine feature maps by summing them, DenseNet combines feature maps by concatenating them. Suitable for tasks requiring strong feature reuse
Vision Transformer [182]	Self-attention mechanism, global modeling	Superior global context understanding	Classification (large datasets)	Requires large amounts of data, future direction. Following the development of ViT, researchers began designing more advanced vision transformers. These include better vision transformer designs, hybrid convolutional neural networks/transformers/multi-layer perceptrons (MLPs), and self-supervised learning.
Swin Transformer [183]	Hierarchical shifted windows Transformer	Global modeling + multi-scale characteristics	Classification, segmentation	Cutting-edge model, for pursuing SOTA performance. Compared to the Transformer, the Swin Transformer offers more features while reducing complexity. It can also conveniently leverage advanced techniques for dense prediction, such as FPN or U-Net.

**Table 6.** Comparison of semantic segmentation architectures

Model name	Core features	Main advantages	Typical medical tasks	Remarks
U-Net [184]	Encoder-decoder, skip connections.	Simple structure, high computational efficiency, suitable for medical data and small-data friendly.	Segmentation lesion detection	Standard baseline in medical segmentation. Use data augmentation to overcome the shortage of medical images.
U-Net++ [185]	Nested dense jump connections, multi-scale feature aggregation, deep supervised training.	Solving the semantic gap problem of U-Net skip connections. Capturing richer multi-scale information. Substantial improvement in accuracy.	Segmentation	It is a systematic improvement on the original U-Net, which typically outperforms it on medical segmentation benchmarks.
Attention U-Net [186]	Gated attention mechanism, spatial attention gate, soft attention mechanism.	Focuses automatically on relevant regions. Improves the performance of small target segmentation. Enhances model interpretability.	Segmentation	Attention mechanisms are particularly well-suited to medical images with large variations in target size and complex backgrounds.
nnU-Net [187]	Automated machine learning framework	“Out-of-the-box”, top-tier performance	Segmentation	Reduces parameter tuning, quickly obtains optimal results.
DeepLab v3+ [70]	Atrous convolution, multi-scale (ASPP)	Accurate boundaries, multi-scale processing	Segmentation	Performed well in bladder cancer segmentation, IoU 0.833, binary accuracy 0.951. Good performance in complex scenes

### Future focus areas

AI cystoscopy technology is advancing to improve diagnostic accuracy and recognition capabilities. The BSU model generates prediction confidence maps by distinguishing between aleatoric and epistemic uncertainties, which provides a critical guarantee of the model’s reliability in real clinical environments [76]. Attention mechanisms, such as the mixed attention module (MAM) and the guided fusion attention structure (GFAS), greatly enhance the model’s capacity to detect minute lesions and subtle abnormalities, resulting in superior Dice and mean intersection over union (MIoU) metrics for multi-scale cancer recognition [74]. Meanwhile, multi-scale feature fusion strategies can effectively address cancer lesions of different sizes and morphologies. Studies show that, even with a small dataset of just 453 images, multi-feature fusion and stacked ensemble learning methods can achieve highly precise identification of NMIBC of stage Ta [77].

## Applications of AI in Bladder Cancer Molecular Subtyping and Drug Development

### Current applications and model construction of AI in molecular subtyping

AI demonstrates emerging potential in predicting molecular subtypes of bladder cancer through routine histopathological analysis, offering a cost-effective and widely accessible adjunct to conventional molecular testing [63]. For instance, a ResNet50-based model achieved an AUROC of 0.85 in discriminating basal-like from luminal-like transcriptional subtypes using H&E-stained WSIs, surpassing the performance of junior pathologists and maintaining robustness across external staining batches [78]. Similarly, a multiple-instance learning-based convolutional neural network (MIL-CNN) model attained an AUROC of approximately 0.90 while providing interpretable regions of interest via class activation mapping, which may offer morphological clues to inform subsequent targeted therapy decisions [63].

In predicting fibroblast growth factor receptor 3 (FGFR3) mutations—a key biomarker for eligibility for targeted therapy (e.g., erdafitinib)—an AI system based on the ShuffleNet architecture significantly outperformed urological pathologists using a predefined visual scoring system for mutation-associated histologic patterns, with AUROCs of 0.701 and 0.725 in 2 independent cohorts [79]. Furthermore, the application of a large-scale, multi-instance learning model to 1,224 cases achieved a sensitivity of 93.6% (with a corresponding specificity of 60.2%) when identifying FGFR3-altered cases within the The Cancer Genome Atlas (TCGA) MIBC/NMIBC cohort [22].

Technically, such models often integrate graph neural networks to model gene interactions, utilize multi-instance learning frameworks for WSI analysis, and employ ensemble methods to enhance generalization [20]. Although not yet a replacement for gold-standard molecular assays, AI-assisted histopathology represents a promising, efficient tool for preliminary molecular stratification in clinical practice [63,78,79], with potential for cost-effective triage [22].

Translating these models into a reliable clinical tool hinges on addressing several underlying bioinformatics challenges, particularly the high dimensionality and technical noise inherent to omics and imaging data. A typical workflow begins with batch effect correction, as variations in tissue fixation, staining protocols, or sequencing platforms can easily mask true biological signals. Methods such as Harmony or Seurat robust principal components analysis (RPCA) have been systematically benchmarked for image-based profiling data, with Harmony consistently ranking among the top performers across multi-lab and multi-instrument scenarios while maintaining computational efficiency [80]. Feature selection alleviates overfitting by reducing the number of features in high-dimensional data, where the number of features far exceeds the number of samples. Information-theoretic methods, such as minimum redundancy maximum relevance (mRMR), effectively analyze gene data by maximizing relevance and minimizing redundancy. However, traditional methods can only capture low-order feature interactions and exhibit unstable performance. The copula entropy-based feature selector (CEFS) and its extended version, copula entropy-based feature selector plus (CEFS+), are capable of capturing full-order feature interactions. Evaluations on various classifiers and datasets demonstrate that this method outperforms mainstream algorithms, particularly excelling on high-dimensional gene data [81]. For scRNA-seq data specifically, graph autoencoder-based methods like single-cell graph autoencoder (scGAE) have been shown to preserve topological structure among cells better than conventional approaches, outperforming other deep learning methods for both trajectory inference and clustering tasks [82]. In the context of single-cell data, an additional complication arises from dropout events, where technical zeros distort the apparent expression of low-abundance transcripts. Imputation methods like Markov affinity-based graph imputation of cells (MAGIC) or single-cell variational inference (scVI) can recover some of this missing structure, although a systematic evaluation of 8 prominent algorithms revealed that while scVI performs competitively for recovering cell population fractions, many methods substantially overestimate the proportion of expressing cells and require careful thresholding to avoid spurious conclusions [83]. When these preprocessing steps are omitted or applied uncritically, model performance on internal validation sets tends to overestimate real-world generalizability.

### Computational workflow for molecular subtyping

Molecular subtyping and immune microenvironment analysis typically center on scRNA-seq, with a standard computational workflow including QC, normalization, scaling, dimensionality reduction via principal components analysis (PCA), batch effect correction, clustering and visualization [e.g., Uniform Manifold Approximation and Projection (UMAP)], cell annotation, identification of differentially expressed genes, and pathway enrichment analysis [84–86]. In bladder cancer research, the Seurat toolkit is commonly used to complete this workflow, and the Harmony algorithm is employed for cross-sample/cross-cohort integration to eliminate technical batch effects and center differences while preserving biological heterogeneity [84–86]. For example, when constructing a multi-center single-cell atlas of bladder cancer, dynamic learning of QC thresholds is performed, with total RNA counts, number of detected genes, and mitochondrial gene proportion as screening criteria. Subsequent normalization is followed by selection of highly variable genes for PCA. On this basis, sample, patient origin, and cohort origin are input as covariates into Harmony for correction, and an unsupervised cluster is achieved by constructing a K-nearest neighbors (KNN) graph using the top 30 principal components [86]. Additionally, some studies have adopted canonical correlation analysis (CCA) and mutual nearest neighbor (MNN) for data integration to address batch effects in multi-cohort scRNA-seq data [87].

#### Feature selection and dimensionality reduction

Feature selection and dimensionality reduction of high-dimensional omics data are critical steps for reducing noise and improving model generalizability. In single-cell data analysis, highly variable genes (e.g., the top 2,000 or 3,000) are typically first identified to focus on biological variation signals [84,85]. Subsequently, PCA is used for linear dimensionality reduction, while UMAP or t-SNE is employed for low-dimensional embedding and visualization, facilitating the interpretation of tumor heterogeneity and cellular subpopulation structure [84,85,87]. For bulk RNA-seq data, cross-platform integration (e.g., combining microarray and RNA-seq cohorts) commonly employs ComBat (based on an empirical Bayes framework) is commonly used to correct batch effects between platforms, combined with UMAP to display high-dimensional transcriptomic heterogeneity [88].

#### Differential analysis and pathway enrichment

After clustering and cell annotation, differential expression analysis is used to identify cluster-specific marker genes and functional states. The FindMarkers function in Seurat, combined with multiple testing correction [e.g., Benjamini–Hochberg false discovery rate (FDR)], can effectively identify differentially expressed genes. Further, tools like clusterProfiler can conduct Gene Ontology (GO)/Kyoto Encyclopedia of Genes and Genomes (KEGG) pathway enrichment analysis to reveal functional associations with cancer-related pathways such as epithelial–mesenchymal transition (EMT), phosphoinositide 3-kinase (PI3K), and Wingless-related integration site (WNT) [84]. Additionally, methods like area under the ROC curve for cells (AUCC) scoring can be used to compare the activity of programmed cell death-related pathways and quantify the functional deviation status of different cell subpopulations [84].

#### Trajectory inference and regulatory network inference

To characterize tumor evolution and cell state transitions, pseudotime trajectory reconstruction is widely applied to single-cell data to visualize dynamic processes such as urothelial cell state transitions [84]. Concurrently, the single-cell regulatory network inference and clustering (SCENIC) framework can infer transcription factor-target gene modules (regulons) and their activity at the single-cell level, thereby identifying key transcriptional regulatory programs driving phenotypic diversity [84].

#### Bulk multi-omics integration and graph-based classification

While the preceding sections focus on single-cell transcriptomic analysis, the following discusses integration of multiple bulk omics layers (e.g., gene expression, copy number, miRNA, and methylation) from the same patient cohort—a distinct analytical paradigm aimed at prognostic stratification rather than cellular deconvolution.

Multi-omics integration can be categorized into horizontal integration (cross-cohorts within the same omics) and vertical integration (multi-layer omics within the same sample). The latter is more aligned with the needs of mechanistic interpretation and target discovery in drug development [89,90]. Vertical integration methods include matrix decomposition [e.g., non-negative matrix factorization (NMF) and multi-omics factor analysis (MoFA)], similarity network fusion (SNF), multi-kernel learning (MKL), ensemble methods [e.g., data integration analysis for biomarker discovery using latent variable approaches for omics studies (DIABLO)], and deep learning models [89,90]. Among these, multi-omics graph convolutional networks (MOGONET), a supervised multi-omics classification algorithm, utilizes graph convolutional network (GCN) to learn view-specific features of each omics and employs view correlation discovery network (VCDN) for cross-omics association modeling, making it suitable for multi-omics classification and stratification prediction [89]. Additionally, autoencoders have been used to integrate multi-omics features such as gene expression, copy number variations, miRNA expression, and DNA methylation, followed by ML algorithms like random forests, k-NN, and AdaBoost for risk stratification and key biomarker mining [89].

#### ML model selection and overfitting control

When constructing prognostic or efficacy prediction models, attention must be paid to model selection and overfitting control. Systematic reviews indicate that feature selection for multi-omics data can employ methods such as Boruta, SVM–recursive feature elimination (RFE), and prediction analysis for microarrays (pamr) to screen key genes/proteins/metabolites and reduce computational burden [89]. Regression-based methods like least absolute shrinkage and selection operator (LASSO) (L1 regularization) and Ridge (L2 regularization) have advantages in high-dimensional data: LASSO tends to sparsely select features by compressing irrelevant variable coefficients to zero, while Ridge shrinks all variable coefficients, making it more suitable for handling highly correlated variables and maintaining stability [85,89]. For survival analysis, algorithms such as CoxBoost, stepwise COX (stepwise Cox proportional hazards regression), and plsRcox can be used to build prediction models related to survival time, combined with external validation sets to assess generalizability [89]. In a bladder cancer multi-omics study, although random survival forests showed extremely high performance on the training set, they exhibited substantial performance degradation on the test set, indicating potential overfitting. Consequently, researchers ultimately favored Ridge regression and explored LASSO-COX combinations to balance variable selection with the interpretability of survival prediction [85].

### AI applications in bladder cancer drug development

AI is being integrated across the drug development pipeline for bladder cancer, enhancing efficiency from patient screening to therapeutic design [91–93].

In clinical trial recruitment, deep learning models applied to H&E-stained WSIs can prescreen patients for molecular eligibility. For instance, one algorithm validated in a cohort of 350 patients with advanced urothelial carcinoma achieved an AUROC of 0.75 with 88.7% sensitivity and 31.8% specificity [92]. This approach could reduce the need for confirmatory molecular testing by 28.7%, thereby lowering trial screening costs [92].

For predicting treatment response, AI frameworks leverage molecular subtyping to forecast outcomes. Researchers have constructed a fibroblast-related gene index combined with CD8^+^ T cell data to predict prognosis and immunotherapy response [93]. Moreover, AI can identify established transcriptional subtypes, such as immune and basal types, and has been used to associate them with survival benefits from atezolizumab, highlighting its utility in patient stratification for targeted therapies [91].

In the realm of novel target discovery and drug design, ML algorithms help pinpoint subtype-specific targets like DAD1, CYP1B1, and REXO2, with subsequent experimental validation advancing targeted strategies [94,95]. Computational tools also facilitate the design and optimization of therapeutic peptides by predicting peptide–receptor interactions, accelerating the early drug discovery pipeline [96].

In summary, AI contributes to cost-effective patient stratification [92], biomarker-driven efficacy prediction [91,93], and data-guided therapeutic design in bladder oncology [91,94,95]. Its full translational potential, however, awaits further prospective clinical validation [94,95].

Integrating multi-omics data for drug response prediction is rarely effective when features from different layers are simply concatenated. Each omic layer—mutations, copy number alterations, gene expression, and protein abundance—carries its own noise structure and statistical properties, so a naive merge tends to dilute weaker signals. A more principled approach uses SNF, which constructs patient similarity graphs from each omic layer independently and then iteratively merges them through message passing across networks [97]. As the noise level increases, LRAcluster still maintains stable performance [average normalized mutual information (NMI) of 0.89]. This indicates that its dimensionality reduction strategy based on low-rank approximation can effectively suppress noise and retain the main structural features of the data, and its explicit matrix decomposition mechanism may play a key role in this process [98].

## Application of AI in Patient-Derived Organoid Models of Bladder Cancer

The integration of AI with patient-derived bladder cancer organoids is advancing translational research primarily by enhancing the models themselves [99,100] and interpreting the complex data they generate [101,102]. This convergence is progressing through several key technical and analytical avenues.

Technically, deep learning architectures, particularly CNN, enable automated, high-precision segmentation and phenotypic quantification of organoid images, forming the basis for high-throughput drug screening platforms [103]. Beyond image analysis, ML frameworks integrate multi-omics data from organoids to build predictive models of individual patient drug response [104].

In predictive modeling, specific AI approaches have demonstrated clinical potential. For instance, a graph neural network-based model [multiview multi-scale graph attention network (MVMSGAT)] was developed to predict responses to neoadjuvant therapy in bladder cancer, showing robust performance (AUROC: 0.8724 ± 0.0511) in validation studies [105].

In immunotherapy research, this integration is creating novel *in vitro* platforms. Systems coculturing organoids with autologous tumor-infiltrating lymphocytes (TILs), combined with AI-driven analysis of immune-cell-mediated killing and spatial interactions, are being developed to model the tumor-immune microenvironment and stratify patients based on predicted efficacy of immunotherapies [99,106].

In data analysis, AI extracts insights from high-dimensional organoid data [101,102]. Computational imaging allows for label-free, single-cell tracking to reveal spatiotemporal tumor heterogeneity [102], while deep learning facilitates multimodal fusion of single-cell multi-omics data (e.g., DNA, RNA, and proteins) [101]. Applied to drug response, these analyses show that mutation profiles partially correlate with treatment resistance—a finding that can be corroborated *in vitro* [107].

Despite these advances, clinical translation faces persistent challenges. Limited automation and a lack of standardized protocols hinder reproducibility and scalability [103]. The inherent absence of a complete tumor-immune microenvironment and systemic signals *in vitro* constrains predictive accuracy for immunotherapy outcomes [108]. Furthermore, high costs and inadequate reimbursement frameworks impede broader clinical adoption and validation [108].

## Application of AI in Bladder Cancer Prognosis

The application of AI in bladder cancer prognostics represents a paradigm shift, enabling a move beyond the static nature of conventional prognostic models [e.g., risk stratification was performed using the European Organisation for Research and Treatment of Cancer (EORTC) and Club Urológico Español de Tratamiento Oncológico (CUETO) scoring systems] by synthesizing high-dimensional and multimodal data [91,109,110]. This integrative capability is a critical advantage. For instance, deep learning models like DeepSurv, which amalgamate diverse clinicopathological variables, have achieved a concordance index (C-index) exceeding 0.88 for predicting disease progression, markedly outperforming traditional risk scores [110].

A key strength of AI lies in its analysis of unstructured data, particularly histopathological WSI. AI models integrate visual pathological information from H&E and immunohistochemical stains by applying multi-instance learning frameworks to WSI, enabling them to predict treatment response with high granularity [111]. Notably, such approaches have demonstrated an accuracy of 84.1% in forecasting response to BCG immunotherapy, with specificity reaching up to 100% for identifying patients with progressive disease [111]. Performance is further enhanced through multimodal fusion; models combining these digital pathology features with clinical parameters have achieved an AUROC of 0.76 for 5-year recurrence prediction, markedly surpassing unimodal approaches [112].

This predictive power translates directly into potential utility at specific clinical decision points [91,111,113]. AI-driven analysis of transcriptomic, genomic, and digital pathology data can stratify patients’ likely benefit from targeted therapies like PD-L1 inhibitors [91]. Similarly, in neoadjuvant therapy, convolutional neural networks (e.g., ResNet-50) analyzing pretreatment histology can predict pathological response to chemoradiotherapy, with reported sensitivity and specificity of 84.6% and 79.2%, respectively [113]. Collectively, these applications underscore AI’s emerging role in enabling personalized and dynamic risk assessment [91,111–113].

## Application of AI in the Development of Novel Medical Devices for Bladder Cancer

### AI-enhanced cystoscopy

The integration of AI, primarily through deep learning-based computer vision, into cystoscopic imaging (white-light and narrow-band) aims to improve the detection and characterization of bladder tumors [17,70]. This technology analyzes endoscopic images or video streams in real time to assist urologists by increasing diagnostic sensitivity—particularly for flat lesions (CIS)—and reducing inter-observer variability [65]. Promising systems include CystoNet, CystoNet-T, CAIDS, and RGB-based algorithms [15–18]. However, prospective clinical validation remains limited. Current clinical trials (NCT07144319) are exploring combinations of AI with enhanced imaging modalities [114]. A critical gap is the need for large-scale studies that integrate AI outputs with comprehensive clinical data to establish robust evidence for its impact on clinical outcomes, rather than purely detection metrics [115,116].

### AI-driven surgical robotics

AI is a key enabler of the evolution of surgical robots, shifting them from teleoperation toward task automation and autonomous assistance in surgery [117]. Current applications focus on specific, supervised tasks, such as analyzing endoscopic images for diagnosis, planning surgery, intraoperative navigation, and guiding instruments [118,119].

The transition to higher levels of autonomy presents a number of substantial challenges. The primary challenge stems from the severe scarcity of publicly available, annotated surgical video data and the persistent performance gap between simulation and real-world scenarios [118,119]. Beyond data, critical safety and robustness issues must be addressed, particularly the need for systems to handle rare intraoperative scenarios (“long-tail” problems) [117]. This requires explainable reasoning, safe fallback strategies to human control, and the ability to adapt continuously [117]. Furthermore, successful clinical integration requires interpretable models to establish trust in surgical procedures, as well as a dedicated framework to facilitate effective human–robot collaboration [120]. Ultimately, it is essential to overcome these interconnected challenges in order to advance beyond the current phase of partial, task-specific automation.

### AI in radiotherapy planning

In radiotherapy for bladder cancer, AI is primarily used to automate processes and improve the accuracy of treatment planning, particularly in the delineation of target volumes [121]. CNN can achieve high geometric agreement (Dice similarity coefficient of 0.93) when automatically contouring the entire bladder, which could potentially streamline workflows [121]. A promising clinical application is in online adaptive magnetic resonance-guided radiotherapy (MRgRT), where AI can enhance efficiency and consistency and enable tighter planning target volume (PTV) margins [122]. This reduces radiation exposure to adjacent healthy tissues [121,122]. However, key limitations remain: Auto-contouring accuracy for boost target volumes is suboptimal; performance is sensitive to large variations in bladder filling; and generalizability to atypical anatomies is limited [122,123]. Consequently, current clinical practice for boost treatments for bladder cancer typically incorporates a semi-automated workflow involving mandatory physician review [123]. Future development will focus on overcoming these contouring challenges to achieve fully automated, robust adaptive radiotherapy [122,123].

## Effect of AI Decision Support Systems on Physicians’ Diagnostic Accuracy in Bladder Cancer

### AI-assisted enhancement of diagnostic accuracy

Emerging data indicate that AI decision support system (AI-DSS) improves diagnostic sensitivity for bladder cancer. The precision urine cytology AI solution for muscle invasion (PUCAS-M) achieved an AUROC of 0.857, significantly outperforming radiologists (AUROC 0.773); integration with clinician assessment (mPUCAS-M) improved sensitivity from 63.9% to 83.3% [124]. A fully automated AI system for predicting histologically high-grade urothelial carcinoma achieved a sensitivity of 63%, superior to board-certified cytopathologists (46%) [51]. In a large-scale multicenter diagnostic study, the cystoscopy AI diagnostic system (CAIDS) demonstrated high accuracy for bladder cancer detection, achieving an accuracy of 0.939 and sensitivity of 0.954 when compared directly with urologists, with a diagnostic latency of only 12 s. In independent external validation sets, the system reached accuracies ranging from 0.978 to 0.991 [17]. Deep learning models achieved 91.2% accuracy in classifying cystoscopic lesions [70]. Furthermore, baseline MRI-based automated ML frameworks can serve as a powerful noninvasive preoperative differentiation tool, reliably distinguishing urothelial carcinoma with squamous differentiation from pure urothelial carcinoma in patients with MIBC, with robust diagnostic performance (AUROC = 0.932 in the test set), and may effectively assist clinicians in preoperative histologic variant diagnosis and individualized treatment decision-making [125].

### Human–AI interaction and complementarity

Recent evaluations of AI in urologic oncology highlight a complementary rather than substitutive role for large language models (LLMs) and multimodal LLMs in clinical practice. In a real-world multidisciplinary tumor board study, ChatGPT-4o achieved only moderate agreement with expert decisions (Cohen’s kappa = 0.61), with full concordance in 56.1% of cases; performance was acceptable in standardized scenarios like bladder tumors and radiotherapy but faltered in complex contexts such as metastatic prostate cancer due to misclassification of tumor burden or treatment sequencing errors [126]. Similarly, a comparative evaluation of multimodal LLMs for cystoscopic image interpretation found that while OpenAI-o3 performed well on anatomical identification (84.5%) and findings description (76%), its performance dropped substantially for higher-order tasks such as lesion reasoning (52.5%) and final diagnosis (48.2%); the model achieved 73.5% accuracy for biopsy indication but only 62.8% for malignancy determination, with in-context learning providing marginal improvements [127]. Collectively, these findings suggest that current AI systems are best positioned as decision support tools that enhance access to standardized care and augment clinical efficiency, whereas human experts remain essential for managing diagnostic uncertainty, complex differentials, and final treatment decisions.

## Human Factors in Clinical AI Applications

Clinical applications of AI face human-related issues such as automation bias, algorithmic dependence, algorithmic aversion, alert fatigue, and skill atrophy. Automation bias and algorithmic aversion refer to excessive trust in AI recommendations and undue rejection of them, respectively; together, they constitute the core risks of human–machine collaboration. Alert fatigue stems from diminished responsiveness caused by high-frequency, low-value alerts and may undermine the safety benefits of AI systems; skill atrophy manifests as a decline in clinical judgment and operational capabilities following long-term reliance on AI, a phenomenon particularly pronounced in training settings. Furthermore, the effectiveness of “human-in-the-loop” as a standalone safety measure has been questioned; relying solely on clinician oversight is insufficient to manage complex, high-volume AI outputs, necessitating more robust governance frameworks and process designs to mitigate potential hazards arising from human factors [128–130].

### Automation bias and algorithm aversion

#### Definition and mechanisms of automation bias

Automation bias refers to clinicians delegating cognitive authority to AI systems and blindly adopting their outputs without fully assessing the applicability of the recommendations [128]. This phenomenon is particularly dangerous in time-sensitive, decision-complex environments; for example, in settings such as intensive care units, the incidence of automation bias increases with the complexity of decision-making [128]. Even when AI systems make errors, humans may overlook their own professional judgment due to trust in the system’s authority, leading to misdiagnoses or delays in treatment [128,131].

#### Manifestations and causes of algorithm aversion

In contrast to automation bias is algorithm aversion, which refers to the tendency of users to ignore or reject AI recommendations even when they are correct [128,132]. This phenomenon may cause healthcare institutions to miss out on the potential benefits of AI; particularly in human–machine systems that require interdisciplinary collaboration and continuous optimization, algorithm aversion can impact the system’s overall effectiveness [132]. Research indicates that algorithm aversion is often associated with factors such as perceived lack of transparency, a lack of a sense of autonomy, and social norms; therefore, improving the system’s explainability and user engagement can help mitigate such issues [133].

#### Empirical research and case studies

Multiple experimental studies have confirmed the existence of algorithmic bias and its impact on clinical decision-making. For example, in a radiology study, highly trained radiologists, upon receiving AI outputs that contradicted their own conclusions, excessively questioned their own judgments and even reversed what were originally correct diagnoses [131]. Another study showed that when AI provided incorrect recommendations, some participants performed worse than when they were not assisted by AI, indicating that AI “advice” may override physicians’ original sound judgments [129]. These findings suggest that post hoc review alone is not effective in preventing automated bias; preventive measures must be implemented at the system design level [128,129].

### Alarm fatigue

#### The concept and consequences of alarm fatigue

Alarm fatigue refers to the phenomenon in which healthcare professionals become desensitized to warning signals due to the frequent receipt of a large number of alarms, leading to reduced response rates or delayed reactions [134]. In clinical practice, alarm fatigue not only increases the workload but also may pose serious safety risks, such as missing critical changes in vital signs or clinically significant abnormal test results [134]. Particularly in intensive care settings, healthcare professionals may face more than 1,000 alarms per shift, making them highly susceptible to the effects of fatigue [134].

#### Contributing factors and management strategies

The primary causes of alarm fatigue include an excessive number of alarms, repetitive triggers, and a high proportion of low-priority events [134]. To reduce alarm fatigue, system design should focus on distinguishing truly urgent situations from routine alerts to avoid unnecessary interruptions [134]. At the same time, measures such as setting customizable notification thresholds can help users identify which alarms are more clinically relevant [133,135]. Furthermore, regularly reviewing and optimizing alarm rules is a crucial means of maintaining system effectiveness [133].

### Deskilling

#### Manifestations of skill degradation

Skill degradation (deskilling) refers to the trend in which healthcare professionals’ practical skills and professional knowledge in certain areas gradually diminish as AI technology becomes widely adopted [132,136]. This includes not only specific clinical procedural skills but also the ability to perform diagnostic reasoning independently [136]. If an AI system fails or its performance declines, physicians without relevant experience may struggle to quickly revert to a state without AI support, thereby compromising patient safety [136].

#### The importance of education and training

To prevent deskilling, it is essential to strengthen continuing education and skills training for healthcare professionals to ensure that they maintain the necessary clinical competencies while using AI [132,136]. On the one hand, physicians should be encouraged to set aside time for “AI-free” practice in their daily work to reinforce foundational skills [132,136]; on the other hand, critical thinking and independent decision-making abilities can be strengthened through simulation exercises, case discussions, and other methods [130]. Furthermore, establishing effective feedback mechanisms to promptly identify and correct cognitive biases resulting from overreliance on AI is also key to ensuring long-term clinical quality [130].

### Limitations of the “clinician-in-the-loop” approach

#### Real-world challenges of the supervised model

Although regulators and developers widely promote the “clinician-in-the-loop” approach as the final line of defense for AI safety, this model encounters numerous limitations in practice [129]. First, clinicians typically care for multiple patients simultaneously and make hundreds of decisions daily, making it difficult for them to maintain constant, vigilant oversight of every AI output [129]. Second, many AI systems are highly “black-box” in nature, and most clinicians lack the technical and statistical expertise to deeply understand their internal mechanisms, making effective oversight difficult [129]. Finally, generative AI can produce fluent and seemingly plausible explanations that mask factual errors, further complicating verification [129].

#### The need for a new governance framework

Given that “clinician in the loop” is not a panacea, it is necessary to establish a more robust governance system to compensate for the shortcomings of individual oversight [129]. This includes establishing interdisciplinary AI governance committees, conducting ongoing algorithm audits, and adopting participatory design methods to ensure that systems align with actual workflows [129]. At the same time, emphasis should be placed on user experience and interface design to make AI outputs easier to understand and verify, thereby reducing the risk of misuse [129]. Furthermore, accountability must be clearly defined to avoid shifting all legal liability onto frontline physicians [129].

### Summary

Overall, the successful application of AI in clinical practice depends not only on its diagnostic accuracy but also on how effectively a series of human-factor issues arising from its use are addressed [128,132]. Future research and practice should focus on the following areas: first, strengthening human–computer interaction design to improve system usability and interpretability; second, improving education and training systems to cultivate healthcare professionals’ AI literacy and critical integration skills; third, establishing robust monitoring and feedback mechanisms to promptly identify and correct issues such as algorithmic bias, alert fatigue, and skill atrophy; and fourth, advancing policy and regulatory frameworks to provide institutional safeguards for the safe and controlled application of AI [128,129,132]. Only in this way can we truly achieve a complementary synergy between AI and human intelligence, thereby promoting a comprehensive improvement in the quality and efficiency of healthcare services.

## Challenges and Future Directions

### Current main challenges

Multi-center heterogeneity and inconsistent annotation currently prevent the creation of large, high-quality, authoritative datasets, which restricts both training and validation. Models trained at single centers perform poorly across sites, demonstrating poor generalizability [137].

Beyond data heterogeneity, the methodological and reporting quality of existing AI tools for bladder cancer remains low. Many studies lack standardized protocols for data preprocessing, feature selection, and model validation, leading to inflated performance estimates and poor reproducibility [138]. Reporting deficiencies are equally concerning: Key elements such as handling of missing data, definition of the reference standard, blinding procedures, and sample size justifications are frequently omitted [138,139]. Consequently, even when models claim high accuracy, the lack of transparent methodology prevents meaningful comparison across studies and impedes clinical translation.

Deep learning decisions remain opaque, and existing interpretability tools rarely reveal the true reasoning behind them, which erodes clinician trust [140–143]. Clinical integration is further obstructed by limited interoperability with electronic health records and the latency of inference, both of which disrupt workflow [144–146]. Deployment requires high-performance hardware and specialized engineers, increasing costs that are rarely reimbursed by insurers [147]. Finally, unresolved algorithmic bias and ambiguous liability hinder ethical acceptance and equitable deployment of bladder cancer AI systems [148].

Algorithmic bias in bladder cancer AI manifests along multiple dimensions that collectively undermine model generalizability. Demographic bias arises when training datasets systematically underrepresent racial and ethnic minorities; ML models predicting survival after RC exhibit marked demographic skew, with cohorts comprising 91.5% White, 5% Black, and only 2.3% Hispanic patients, resulting in biased performance across sex and racial subgroups [149]. Institutional bias emerges when models are trained on single-center retrospective data, causing them to learn site-specific artifacts rather than robust pathological features of urothelial carcinoma, which critically threatens external validity [150]. The risk of overfitting is exacerbated by class imbalance in rare bladder cancer variants and the tendency of deep learning architectures to memorize majority-class patterns from homogeneous cohorts [151]. Most bladder cancer AI studies rely solely on internal validation without independent multicenter evaluation, leaving model robustness under real-world clinical variability largely unproven [152]. Dataset representativeness is further compromised by socioeconomic disparities, where underserved populations have sparser electronic health records, leading risk-stratification tools to systematically underestimate recurrence probabilities for these patients [153]. Without explicit subgroup performance evaluation using standardized fairness metrics and rigorous multi-institutional external validation, bladder cancer AI systems risk amplifying existing oncological disparities rather than ameliorating them [124].

### Regulatory approval and health technology assessment

In the United States, the FDA has established 2 primary pathways for AI/ML-based devices: the 510(k) premarket notification and the *De Novo* classification [154]. A systematic review of 950 FDA-authorized AI/ML devices found that 97% were cleared via the 510(k) pathway, with only 5% undergoing prospective testing and 8% including human-in-the-loop evaluation [155]. Notably, 95.5% of device summaries failed to report demographic information, and only 1.6% reported data from randomized clinical trials [154]. These gaps in clinical validation underscore the need for stronger oversight of AI devices before market authorization [154].

International regulatory perspectives are also evolving rapidly. In the European Union (EU), the Artificial Intelligence Act [AI Act; Regulation (EU) 2024/1689] introduces a risk-based classification system that classifies AI systems used for medical purposes as high-risk, imposing stringent requirements including conformity assessments, technical documentation, human oversight, and post-market monitoring [156]. Concurrently, the Medical Device Regulation (MDR; 2017/745) and *In Vitro* Diagnostic Regulation (IVDR; 2017/746) govern the safety, performance, and clinical evaluation of AI-enabled devices and diagnostics, respectively, emphasizing quality management systems and post-market surveillance [157]. In China, the National Medical Products Administration (NMPA) has established a comprehensive regulatory framework for artificial intelligence in medical devices (AIMD), classifying AI-assisted diagnostic or detection software as class III high-risk medical devices and mandating rigorous whole-life-cycle QC, including training data representativeness, algorithm validation, and clinical trial requirements [157]. While harmonization between FDA, EU, and Chinese frameworks remains incomplete, these developments underscore a global trend toward more rigorous oversight of AI/ML technologies in oncology. Collaborative regulatory science initiatives and multinational trials will be essential to ensure that AI innovations in bladder cancer meet diverse jurisdictional standards without duplicative evidence generation [158].

### Clinical workflow integration and implementation barriers

The clinical translation of AI for bladder cancer requires integration into existing hospital workflows. Achieving this goal depends on DICOM- and HL7-based data orchestration that embeds AI outputs directly into picture archiving and communication system (PACS), laboratory information system (LIS), and electronic health record (EHR) systems [137]. In the case of bladder cancer, currently, AI tools have been implemented in 4 ways: real-time cystoscopy, WSI histopathological grading, automated interpretation of urinary cytology, and multimodal clinical decision support (CDS) incorporating EHR [137,151]. AI-assisted diagnostic results should be generated in a timely manner to serve as a reference for clinicians. However, if clinicians have already completed their diagnosis, the value of the AI results as a reference would be diminished. Currently, we should not let the AI make decision alone; non-editable results in one direction promote automation bias [137,140].

A survey of 63 U.S. pathologists revealed that most laboratories would have substantial difficulty providing AI-guided image analysis today; the main barriers include high implementation costs, lack of reimbursement, and integration procedure [159]. Among laboratories experienced with computational pathology, the challenges included lack of interoperability across platforms and discordant outputs from similar algorithms [159]. Financial sustainability is also a problem that cannot be neglected. A European study evaluating digital pathology implementation found that while the technology achieves cost recovery within 6 years, high upfront costs for scanners, training, and system integration pose major barriers, particularly for smaller institutions [160].

### Standardized datasets, open challenges, or benchmarking initiatives

The lack of publicly available, high-quality, and representative standardized datasets represents a fundamental barrier to fair evaluation and meaningful comparison across bladder cancer AI studies. Pathology AI reviews emphasize that ideal benchmark datasets should adhere to the FAIR principles (findable, accessible, interoperable, reusable) and reflect real-world “in-the-wild” data distributions and population coverage [161]. The research community should embrace open science practices, including transparent sharing of data, code, and trained models, as reproducibility in medical AI depends critically on these practices, and recommends benchmarking new models against established clinical prediction tools—such as the EORTC NMIBC risk calculator—and previously published models [152].

Recent bladder cancer studies further highlight this lack of standardization. In a human epidermal growth factor receptor 2 (HER2) scoring AI study for urothelial bladder cancer, researchers had to rely on transfer learning from a breast cancer model due to the absence of large-scale bladder cancer datasets, and manual annotation of approximately 100,000 cells from only 22 slides was required [162]. Interobserver agreement among pathologists was only moderate (kappa = 0.48), and no disease-specific HER2 scoring standard exists for bladder cancer [162]. Similarly, in bladder cancer cell sheet modeling, different cell lines (T24 versus 5637) showed inconsistent sheet-forming capabilities, indicating a lack of standardized protocols and reference benchmarks across laboratories [163]. These examples underscore the urgent need for public, multi-center datasets and community-agreed benchmarking frameworks in bladder cancer research.

The core solution path for the lack of a unified benchmarking framework in AI research for bladder cancer focuses on establishing public, standardized, multi-center real-world datasets as benchmarking platforms, accompanied by mandatory transparent reporting and external validation paradigms. Existing evidence shows that open challenges can temporarily promote data sharing and method comparison, but more sustainable approaches include long-term accessible multi-center open datasets combined with federated learning baseline evaluations, as well as the adoption of internationally recognized outcome definitions and evaluation systems benchmarked against existing clinical tools in the field of prognosis prediction [152,164,165].

Some previous federated learning medical imaging datasets, released through challenges, have become inaccessible post-event, leading to difficulties in replication and ongoing comparison [164]. Therefore, open challenges are better positioned as stage-specific methodological catalysts, while long-term benchmarking requires data with long-term accessibility and reproducible baselines [164]. Additionally, achieving clinical translation requires “creating public multi-center benchmarks” and shifting the validation paradigm from purely retrospective diagnostic accuracy to prospective utility studies (e.g., impact on treatment decisions) [165]. This provides direction for the design of open challenges: Challenge objectives should shift from “boosting AUROC” to interpretable, reproducible, and generalizable clinical utility metrics [165].

### Future development directions

In order to facilitate the clinical translation and application of AI in the treatment of bladder cancer, future research should prioritize several key areas. Firstly, innovation in multimodal fusion is essential, necessitating the development of efficient strategies, such as cross-modal attention mechanisms and knowledge distillation, to integrate radiological and endoscopic imaging with pathology and genomics [166,167]. Furthermore, enhancing model interpretability through advanced technologies and inherently interpretable architectures is crucial for building clinical trust and transparency [168]. Additionally, decentralized ML approaches, such as swarm learning [169] and federated learning [170], enable distributed, multi-center collaboration by addressing data privacy concerns and overcoming data silos.

Beyond these, developing dynamic prediction systems that can integrate longitudinal data will enable continuous assessment of disease progression and treatment response [171]. Moreover, large-scale, multi-center prospective studies are required to validate the clinical utility and cost-effectiveness of AI models [172,173]. In line with CONSORT-AI [172] and Standard Protocol Items: Recommendations for Interventional Trials–Artificial Intelligence (SPIRIT-AI) [173] guidelines, clinical trials must clearly describe the AI intervention—including its functionality, integration environment, input/output processing, and human–AI interaction—along with an analysis of error cases. Finally, advancing medical AI requires enhanced evidence-based regulation, robust post-market surveillance, a focus on clinically meaningful endpoints, and the engagement of key stakeholders [174].

## Conclusion

In summary, AI is reshaping the paradigm of precision oncology in bladder cancer. By synthesizing multimodal data, it offers substantial potential to augment clinical workflows—from imaging and histopathology analysis to treatment personalization and prognostic stratification—thereby facilitating individualized patient management.

Moving forward, key challenges pertaining to data heterogeneity, model interpretability, and seamless clinical integration must be addressed. Interdisciplinary efforts focused on robust validation, standardization, and ethical frameworks will be crucial to translating these technological advances into safe, effective, and scalable solutions that ultimately improve patient outcomes.

## Ethical Compliance

As this work is a comprehensive review of existing literature and does not involve any original research with human participants or animals, no ethics approval was required.
