# Supplementary material for: The Current Status and Evaluation of Artificial Intelligence in Bladder Cancer: Clinical Applications and Research
Source: Comput Struct Biotechnol J. 2026 Oct 19;35(1):0197. doi: 10.34133/csbj.0197 (PMC13678086; doi:10.34133/csbj.0197)
Supplement: Supplementary file 1 [file csbj.0197.f1.pdf]

Supplementary Table 1. Evidence-level classification of included studies (N=52).

Research Field	Evidence Level	Model Type	Study Details
**Tumor Imaging**	Level IV	Bladder Cancer-Specific	[3] Taguchi S (2021)
	Level II	Bladder Cancer-Specific	[2] Gong Y (2025)
	Level II	Bladder Cancer-Specific	[37] Du Y (2025)
	Level II	Bladder Cancer-Specific	[39] Girard A (2023)
	Level II	Bladder Cancer-Specific	[41] Sun R (2024)
	Level II	Bladder Cancer-Specific	[44] Wei Z (2024)
	Level II	Bladder Cancer-Specific	[4] Song H (2023)
	Level I	Bladder Cancer-Specific	[26] Hammouda K (2021)
	Level I	Bladder Cancer-Specific	[34] Garapati SS (2017)
	Level I	Bladder Cancer-Specific	[35] Yang Y (2021)
	Level I	Bladder Cancer-Specific	[36] Xiong S (2024)
	Level I	Bladder Cancer-Specific	[38] Lisson CG (2025)
	Level I	Bladder Cancer-Specific	[40] Gresser E (2023)
	Level I	Bladder Cancer-Specific	[42] Cha KH (2017)
	Level I	Bladder Cancer-Specific	[43] Ying T (2021)
**Tumor Pathology**	Level III	Bladder Cancer-Specific	[6] Pan J (2023)
	Level III	Bladder Cancer-Specific	[9] Wu S (2023)
	Level II	Bladder Cancer-Specific	[60] Jiao P (2025)
	Level II	Bladder Cancer-Specific	[8] Lotan Y (2025)
	Level I	General-Purpose	[54] Chen RJ (2024)
	Level I	General-Purpose	[55] Xu H (2024)
	Level I	General-Purpose	[56] Lu MY (2024)
	Level I	General-Purpose	[57] Shaikovski G (2025)
	Level I	General-Purpose	[58] Yan F (2025)
	Level I	General-Purpose	[59] Yang Z (2025)
	Level I	Bladder Cancer-Specific	[5] Tsuneki M (2022)
	Level I	General-Purpose	[61] Xiang J (2025)
	Level I	Bladder Cancer-Specific	[7] Debatin NF (2024)
**Molecular Subtyping**	Level III	Bladder Cancer-Specific	[78] Zheng Z (2025)
	Level III	Bladder Cancer-Specific	[79] Loeffler CML (2022)
	Level II	Bladder Cancer-Specific	[21] Boll LM (2025)
	Level II	Bladder Cancer-Specific	[22] Bannier P-A (2024)
	Level II	Bladder Cancer-Specific	[63] Woerl A-C (2020)
	Level I	Bladder Cancer-Specific	[93] Li J (2024)
	Level I	Bladder Cancer-Specific	[94] Wang Z (2025)
	Level I	Bladder Cancer-Specific	[95] Zhou Z (2025)
	Level I	Bladder Cancer-Specific	[105] Luo X (2024)
	Level I	Bladder Cancer-Specific	[20] Liu Y (2025)
**Cystoscopy**	Level IV	Bladder Cancer-Specific	[15] Shkolyar E (2019)
	Level III	Bladder Cancer-Specific	[17] Wu S (2022)
	Level I	Bladder Cancer-Specific	[16] Jia X (2023)
	Level I	Bladder Cancer-Specific	[18] Yoo JW (2022)
**Prognosis Prediction**	Level II	Bladder Cancer-Specific	[111] Jiang F (2025)
	Level I	Bladder Cancer-Specific	[112] Lucas M (2022)
**Drug Development**	Level II	Bladder Cancer-Specific	[92] Juan Ramon A (2024)
**Radiotherapy**	Level II	Bladder Cancer-Specific	[123] Azzarouali S (2024)
**Organoid**	Level I	General-Purpose	[99] Ao Z (2022)
**Stem Cell Therapy**	Level I	General-Purpose	[96] Lee SB (2024)
**General Methodology**	Level I	General-Purpose	[80] Arevalo J (2024)
	Level I	General-Purpose	[81] Yan X (2025)
	Level I	General-Purpose	[82] Luo Z (2021)
	Level I	General-Purpose	[83] Valyaeva AA (2026)

Supplementary Table1. Evidence-level classification of included studies. The 52 included studies are grouped by research domain and sorted by evidence level within each domain. Evidence levels are color-coded: Level I (red), technical feasibility with internal validation only; Level II (orange), external or multicenter retrospective validation; Level III (blue), observer-based assessment or reader studies; Level IV (green), prospective clinical validation. Model types (bladder cancer-specific vs. general-purpose) are indicated in the "Model Type" column.
